# Is There a Relationship Between Workload and Occurrence of Back Pain and Back Injuries in Athletes?

**DOI:** 10.3389/fphys.2020.00894

**Published:** 2020-07-24

**Authors:** Erika Zemková, Zuzana Kováčiková, Ludmila Zapletalová

**Affiliations:** ^1^Department of Biological and Medical Sciences, Faculty of Physical Education and Sports, Comenius University in Bratislava, Bratislava, Slovakia; ^2^Sports Technology Institute, Faculty of Electrical Engineering and Information Technology, Slovak University of Technology, Bratislava, Slovakia; ^3^Institute of Physiotherapy, Balneology and Medical Rehabilitation, University of Ss. Cyril and Methodius in Trnava, Trnava, Slovakia; ^4^Department of Natural Sciences in Kinanthropology, Faculty of Physical Culture, Palacký University Olomouc, Olomouc, Czechia

**Keywords:** Acute:Chronic Workload Ratio, back problems, individual sports, team sports, training load

## Abstract

The back is subjected to a great deal of strain in many sports. Up to 20% of all sports injuries involve an injury to the lower back or neck. Repetitive or high impact loads (e.g., running, gymnastics, skiing) and weight loading (e.g., weightlifting) affect the lower back. Rotation of the torso (e.g., golf, tennis) causes damage to both, the lumbar and thoracic spine. The cervical spine is most commonly injured in contact sports (e.g., boxing, football). One of the factors that increases the odds of injuries in athletes is excessive and rapid increases in training loads. In spite of currently emerging evidence on this issue, little is known about the balance between physiological loading on the spine and athletic performance, versus overloading and back pain and/or injury in athletes. This scoping review aims (i) to map the literature that addresses the association between the training load and the occurrence of back pain and/or injury, especially between the Acute:Chronic Workload Ratio (ACWR) and back problems in athletes of individual and team sports, and (ii) to identify gaps in existing literature and propose future research on this topic. A literature search of six electronic databases (i.e., MEDLINE, PubMed, Web of Science, SCOPUS, SportDiscus, and CINAHL) was conducted. A total of 48 research articles met the inclusion criteria. Findings identified that fatigue of the trunk muscles induced by excessive loading of the spine is one of the sources of back problems in athletes. In particular, high training volume and repetitive motions are responsible for the high prevalence rates. The most influential are biomechanical and physiological variations underlying the spine, though stress-related psychological factors should also be considered. However, limited evidence exists on the relationship between the ACWR and back pain or non-contact back injuries in athletes from individual and team sports. This may be due to insufficiently specified the acute and chronic time window that varies according to sport-specific schedule of competition and training. More research is therefore warranted to elucidate whether ACWR, among other factors, is able to identify workloads that could increase the risk of back problems in athletes.

## Introduction

Back injuries and back pain are among the most common health problems in athletes affecting their performance (Trompeter et al., 2017). Acute injury or pain can be caused by falling, being tackled in team sports, fighting in combat sports, or while lifting heavy weights (Caine and Nassar, 2005; Aasa et al., 2017; Bromley et al., 2018; Plais et al., 2019). However, much more common than acute incidents are chronic back problems (Alzahrani et al., 2019). In many sports, the back and spinal column undergoes elevated stress for a long time (Lawrence et al., 2006). This may result in inflammation around the vertebrae and back muscles, which sometimes causes injuries to the discs but much more often leads to upper or lower back pain (Trompeter et al., 2017). Sciatica, for instance, is back pain that also affects the back of the legs or even the feet (Koes et al., 2007). It can occur in cyclists who are in a flexed forward posture or athletes of water and swing sports who perform a great deal of trunk rotation. Particularly in sports with repetitive asymmetric loading, the sacroiliac joint dysfunction is a frequent cause of low back pain in athletes (Peebles and Jonas, 2017). In most of these sports, asymmetric loading causes side-to-side dysbalances that may greatly enhance susceptibility to back pain. A clear example is an association between repeated golf swings and golf-related low back injuries (Cole and Grimshaw, 2016). Trunk rotational power represents the measure that reflects this asymmetric loading during trunk rotation (Zemková et al., 2019a). Its values were found to be significantly higher on the dominant than non-dominant side in golfers (∼15%), tennis (∼12%) and hockey players (∼14%) at lower or higher loads, whereas there were no significant between-side differences in a control group of physically active subjects (∼7%). This parameter is also able to reflect the specificity of training programs in the preparatory and competitive periods in canoeists, hockey and tennis players (Poór and Zemková, 2018). Similarly, wheelchair athletes, who suffer from spine curvature disorders, are at greater risk of back problems (Samuelsson et al., 2001). This is related to mainly those participating in sports that involve trunk rotational motions under loading and unloading conditions (Goosey-Tolfrey, 2010). A recent study demonstrated that wheelchair table tennis players exhibit lower mobility in the thoracic and lumbar regions of the spine during trunk flexion as well as lower lumbar inversion and pelvic retroversion than able-bodied athletes (Zemková et al., 2018b). Their limited range of motion (ROM) during trunk rotations and decreased posterior concavity contributes to lower trunk rotational velocity (Zemková et al., 2018b) and it may also increase the risk of spine injury. Velocity of trunk rotational movement is also compromised in older athletes, most likely due to their reduced ROM as a result of increased trunk stiffness with aging (Zemková et al., 2018a).

In spite of the fact that low back pain in athletes is among the most prevalent musculoskeletal condition with incidence rates of 1–30%, it is often neglected in research studies (Graw and Wiesel, 2008). The prevalence of low back pain is 1–94%, (the highest in rowing and cross-country skiing), and its point prevalence is 18–65% (the highest in rowing and the lowest in basketball) (Trompeter et al., 2017). Prevalence rates vary during an athlete’s season with the highest rates observed during the peak season (Newlands et al., 2015). The evidence on risk factors is mostly restricted to lumbar spine and hip flexibility, back muscle strength, trunk extensor/flexor endurance, and some anthropometric characteristics (Moradi et al., 2015; Trompeter et al., 2017). However, less attention has been paid to sport related factors. Among those, high training volume and repetitive motions contribute to the high prevalence rates (Fett et al., 2017). Athletes involved in impact sports are at risk for certain spinal pathologies that are associated with repetitive loading of the spine (Lawrence et al., 2006). For instance, higher incidence rates of spondylolysis and degenerative disk disease have been reported in elite athletes who participate in more intense training over a longer period of time than those who do not (Lawrence et al., 2006). Though muscle fatigue is often a symptom of back pain in athletes, factors like the number of training sessions per week only rarely have been investigated (Fett et al., 2017). The concept that compares the amount of acute workload performed in a 1-week relative to a 4-week chronic workload can provide useful information for designing the optimal workload that would improve athlete performance, and at the same time, decrease the likelihood of back pain and related injuries.

The Acute:Chronic Workload Ratio (ACWR) is a simplified version of the fitness-fatigue model that was first introduced by Banister et al. (1975). It is definied as the ratio between training loads in recent periods (∼5–10 days) and over longer periods (∼4–6 weeks) (Foster et al., 1999; Hulin et al., 2016b). The ACWR is calculated by using the rolling or exponentially weighted moving average model (Hunter, 1986). Though both models are used in practice, the first one is unable to deal with the decaying nature of fatigue and fitness effects over time and the non-linearity of workload and the occurrence of injury; thus it may less precisely reflect variations of accumulated loads (Williams et al., 2017; Menaspa, 2017). The second one, which attributes a decreasing weighting in order to compensate for the load latency effects (Williams et al., 2017), is more sensitive to detect increases in injury risk at higher ACWR ranges during the preseason and in-season periods (Griffin et al., 2020).

These models have been used to better control and understand the training process. Several studies have documented the association between the actual and modeled performance in a variety of individual sports (e.g., Taha and Thomas, 2003; Jobson et al., 2009) or injuries in team sports (e.g., Hulin et al., 2016a; Bowen et al., 2017; Murray et al., 2017). Contrary to this, less attention has been paid to the investigation and use of these models in association with the occurrence of injuries or pain that especially involve the spine and back muscles. So far the training volume, internal and external loads in different types of training, sport-specific demands on the spine in relation to back pain and/or injury have been primarily investigated (e.g., Maselli et al., 2015; Newlands et al., 2015; van Hilst et al., 2015). There is however, no sufficient evidence to determine the relationship between ACWR and the occurrence of back problems in athletes. A question also remains as to whether ACWR can be predictive of an athlete’s risk of back pain and/or injury.

To this end, two main questions were addressed in this scoping review: (1) Is the training load associated with the occurrence of back problems in athletes of individual and team sports? (2) Is there a relationship between the ACWR and back pain and/or injury in these athletes? Accordingly, this scoping review aimed at (i) mapping the literature that addresses the association between the training load and the occurrence of back pain and/or injury, especially between the ACWR and back problems in athletes of individual and team sports, and (ii) identifying gaps in existing literature and proposing future research on this topic.

## Methods

The article was designed as a scoping review (Armstrong et al., 2011). In order to answer the above questions and to identify a gap in existing research in the field, a literature search was provided. Electronic literature was searched on MEDLINE, PubMed, Web of Science, SCOPUS, SportDiscus, and CINAHL databases. Further searches were conducted on Elsevier, SpringerLink, EBSCOhost, and Google Scholar. Besides articles in peer-reviewed journals, also conference proceedings were analyzed. The search was confined to studies closely associated with the major topic of this review, i.e., investigating the relationship between training loads and the occurrence of back problems in athletes, particularly between the ACWR and back pain and/or injury in athletes of individual and team sports. Our primary focus was concentrated on outcome measures of the ACWR and data related to back problems in athletes of individual and team sports. Using this approach, however, led to the identification of a limited number of studies that were able to meet the eligibility criteria for this review. Therefore the search was later widened to include all relevant studies that investigated the relationship between the training load and back pain and/or injury in athletes. Together these help us to identify gaps in the current literature regarding the training load, especially the ACWR and their associations with back pain and/or injury in athletes of individual and team sports, and suggest a proposal for future research.

**FIGURE 1:**
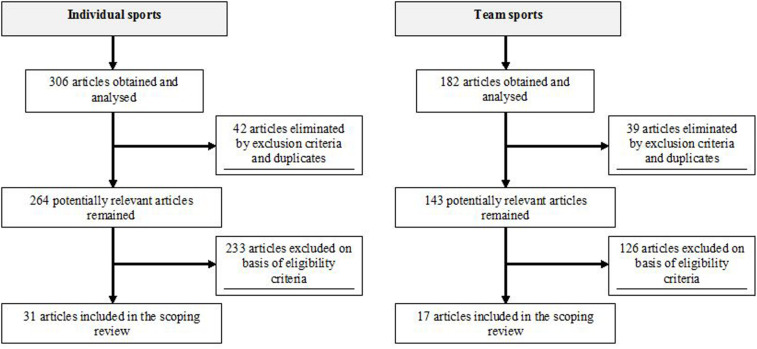
Flow chart illustrating phases of the literature search and study selection.

The target population was athletes of individual (cricket, cycling, dancing, diving, golf, gymnastics, horseback riding, jiu jitsu, judo, powerlifting, rowing, running, skiing, swimming, triathlon, weightlifting, wrestling) and team sports (basketball, florball, handball, hockey, soccer, tennis, volleyball). The most frequent terms “Acute:Chronic Workload Ratio,” “back injuries,” “back pain,” “back problems,” “competition,” “external training load,” “fitness level,” “injury risk,” “internal training load,” “low back pain,” “neck/cervical pain,” “non-contact injury,” “thoracic pain,” and “training” were combined with particular individual and team sports. Additional searches were performed by using the words from subheadings, such as factors contributing to back pain and/or injury in athletes with respect to their localization, intensity, frequency, and duration. The key inclusion criterion was that studies involved athletes of individual and team sports with back pain and/or injury, a specified training program, and objective or subjective measures relevant to this review. Studies were excluded if they were incomplete (abstracts, etc.), not peer-reviewed, did not contain original research, and were not written in the English. Studies that failed to meet these criteria for this review were excluded. Figure 1 represents particular phases of the search process.

## Results and Discussion

### Overview of Studies Dealing With Back Pain and/or Injury in Athletes of Individual Sports

The key data elements that were sought for each study were categorized as follows: (1) study design, (2) study population (number of athletes, sex, age, type of sport, and performance level), (3) training characteristics, (4) low back pain information, (5) diagnostic methods used for identification of lumbar spine, low back pain and lower back injuries, and (6) study outcomes.

Out of the 31 research articles (Table 1), 21 studies (68%) included both sexes, six studies (19%) dealt with male athletes, and four studies (13%) focused on female athletes. A total of 2999 athletes of mean age of 26.1 years was evaluated, out of which 1819 were males and 1180 were females. Fifteen studies were conducted with elite athletes, nine studies assessed non-elite athletes, and six studies were conducted in both elite and non-elite athletes. For the purpose of this review, elite athletes were defined as those competing professionally at national level, international level or at the Olympic Games. Athletes competing at district and regional competition levels, as well as recreational and amateur athletes were defined as non-elite. Also athletes competing at high schools, colleges, or universities were considered as non-elite.

**TABLE 1:** Training characteristics and identified back problems in athletes of individual sports.

**Authors**	**Sport and performance level**	**Subjects**	**Training**	**Back pain and/or injury**	**Diagnostics of back pain and/or injury**	**Main findings**
Baranto et al., 2006	– 20 Swedish elite divers (14 girls and 6 boys), still active in competition – 18 divers active in competitive diving at follow-up	Age at baseline: 16.4 ± 3.1 years Age at follow-up: 21.2 ± 3.0 years	Training days/week at follow-up: 4.0 ± 1.6 h Training hours/week at follow-up: 6.5 ± 2.9 h	Back pain was self-assessed. It was graded as moderate if it does not affect training and competition. If it affects training and competition it was graded as severe. 16 divers with previous or present back pain (first episode at age 15 ± 1.6 years) Causes of back pain: – Diving (8) – Trauma (2) – Do not know (4) – Not answered (2) Pain deteriorated with training (10) Pain improved with training (2)	– MRI examination – Neurological examination of lower extremities and the spine – Back pain questionnaire	– MRI abnormalities were observed in 3 divers at baseline – MRI abnormalities were observed in 4/7 divers trained more than 8 h/week (at follow-up) – MRI abnormalities were also found in 8/11 divers trained less than 8 h/week – High prevalence of back pain and MRI changes was observed; their causal relationship was confirmed – Young age was associated with high frequency of back pain – Growth spurt was associated with high risk of occurrence of degenerative changes of the thoracolumbar spine – Mild structural scoliosis in the thoracic spine in 1 diver – Tenderness in the thoracic in 8 divers – Tenderness in the lumbar spine in 5 divers – 5 divers presented MRI abnormalities and tenderness in the thoracolumbar spine – MRI abnormalities but no tenderness in 5 divers – 5 divers presented tenderness but no MRI abnormalities in the spine – MRI abnormalities or tenderness in 3 divers – Abnormalities in the thoracolumbar spine at baseline MRI examination in 12 out of 18 divers – Abnormalities in the thoracolumbar spine at follow-up MRI examination in 12 out of 17 divers – 20 new abnormalities in the spine were observed at follow-up
Jonasson et al., 2011	– 75 Swedish top male athletes (17 divers, 17 weight-lifters, 11 wrestlers, 13 orienteers, and 17 ice-hockey players) – Control group consisted of 21 non-athletes	The median age of top athletes: 21.5 years The median age of non-athletes: 28 years The median age of divers: 17 years (youngest group)	The median training days/week for divers: 3.5 Training hours/week: 6.5 ± 2.9 h Mean training duration: 8.8 h/week Four divers trained 11 h/week Training intensity was not specified ins other group of athletes.	All athletes and controls with no knowledge of previous or present back symptoms	– Self-assessed pain-oriented questionnaire – The EuroQol questionnaire – The Oswestry Disability Questionnaire	– The frequency of cervical, thoracic and lumbar pain in the last week/last year was 35/55%, 22/33%, and 50/68%, respectively
Kaneoka et al., 2007	– 56 Japanese elite swimmers (35 male and 21 female) – defined as a “high-load group” – 38 students (24 male and 14 female) defined as a “low-load group” or control group.	Age: 15–27 years	High load group Swimming duration: 9.3 years Swimming distance: 49,047 m/week Low load group Swimming duration: 5.4 years Swimming distance: 8440 m/week	– Low back pain in a high load group (yes/no): 43/13 – LBP in a low load group (yes/no): 33/5 – Severe LBP in a high load group (yes/no): 29/27 – Severe LBP in a low load group (yes/no): 14/24	– Questionnaire – MRI examination	– Degenerated disks at various disk levels observed in 38 elite swimmers and 11 controls – The prevalence was significantly greater in elite swimmers – No association was observed between the low back pain, swimming strokes, and disk degeneration – L5-S1 intervertebral segment is the most affected by excessive competitive swimming activities
Schultz and Gordon, 2010	– 66 recreational Australian cyclists aged 18 years and over (49 male and 17 female) – Divided into LBP group (*n* = 33) and NLBP group (*n* = 33)		LBP group Cycling experience: 6.0 ± 11.6 years Distance cycled: 250 ± 131.0 km/week Cycling frequency: 5.0 ± 1.4 days/week NLBP group Cycling experience: 3.0 ± 7.4 years Distance cycled: 150.0 ± 135 km/week Cycling frequency: 4.0 ± 1.8 days/week	33 cyclists (23 males and 10 females) reported LBP during or after cycling within the last 6 months 27 cyclists (26 males and 7 females) reported no low back pain during or after cycling	– Survey by Wilber et al. (1995)	– LBP group cycled significantly more in a week than NLBP group – Training volume of 160 km/week or more was associated with 3.6 times higher incidence of LBP than in cyclists with training volume less than 160 km/week – Training volume less than 160 km/week reduced the risk of new occurrence of LBP in recreational cyclists
Muyor et al., 2011	– 60 Spanish elite male cyclists and 60 master cyclists	Elite Mean age: 22.95 ± 3.38 years Master Mean age: 34.27 ± 3.05 years	Elite Training days/week: 5.5 ± 1.46 Training hours/day: 3.23 ± 0.69 Master Training days/week: 3.23 ± 1.35 Training hours/day: 3.06 ± 1.28	Without spinal pain in 3 months prior study enrollment	– Spinal Mouse	– Elite cyclists present thoracic hyperkyphotic postures in standing position; these changes in spinal curvatures may be a reason for the development of LBP
Ruckstuhl and Clénin, 2019	– 111 Swiss elite cyclists (66 cyclists of endurance disciplines and 45 cyclists of technical disciplines) – Endurance athletes (road cycling, MTB or cyclo-cross): 39 males and 27 females – Technical discipline athletes (BMX, trial, downhill or 4×): 38 males and 7 females – All cyclists – a Swiss national team members	Age of endurance athletes: 19.5 ± 5.8 years Age of technical athletes: 19.6 ± 3.5years	Training hours/week: 11.5 ± 5		– Core strength testing – Back pain questionnaire (modified Oswestry-Disability-Index) – Questionnaire on prior to training in general and core strength training – Sports medical examination – Evaluation of the sitting position on the bicycle	– Back pain in cyclists is frequent – In training – every 3rd suffers slight and every 10th rider moderate to heavy back pain – In competition – half of the cyclists are affected by back pain episodes (22.5% of them have moderate to heavy back pain) – 1/3 of elite cyclists have insufficient core strength – Core strength of the dorsal muscle chain is positively related to a lesser frequency of back pain – No significant association between back pain and core strength of the ventral and lateral chain – 21.6% cyclists are free of pain – 31.5% of cyclists suffer at least once a month of back pain – 37.9% of cyclists have back pain episodes 2–7 days/month – 9% of cyclists suffer from back pain more often – 6% of cyclists suffer from back pain nearly every day – 34.2% of cyclists suffer from slight back pain in training – 9.9% of cyclists suffer from moderate to very heavy back pain in training – 27.9% of cyclists suffer from slight back pain in competition – 22.5% of cyclists suffer from moderate to very heavy back pain in competition – 4.5% of athletes are moderately to heavily limited in daily life due to back pain – Endurance disciplines athletes suffer from back pain more than technical disciplines athletes
Piotrowska et al., 2017	– 167 amateur Polish male cyclists	Age: 31.0 ± 8.0 years Weight: 74.9 ± 9.5 kg Height: 1.8 ± 0.1 m	Training experience: 7.2 ± 6.7 years Training hours/week: 11.8 ± 6.9	Spine pain – experienced by 70 cyclists Lumbar spine pain (33%) – one of the most common pains	– Questionnaire	– Spine pain in 41% of athletes (26% is related to the lumbar spine) – Number of hours devoted to training/week influence the case of spine pain
Murray et al., 2009	– 64 English amateur golfers (43 men and 21 women)	LBP group Age: 56.4 ± 8.4 years Height: 179.1 ± 8.7 cm Weight: 85.2 ± 14.5 kg Male to female: 26:2 Control group Age: 54.3 ± 14.4 years Height: 176.8 ± 7.8 cm Weight: 78.2 ± 10.2 kg Male to female: 32:4	LBP group Rounds/week: 2.0 ± 0.9 Years of play: 27.3 ± 11.3 Control group Rounds/week: 2.1 ± 1.1 Years of play: 22.9 ± 15.6	Only golfers with LBP due to repeated injuries. LBP group (*n* = 28) – golfers with history of LBP within the last 12 months prior to the study enrollment (also currently suffering from LBP) Control group (*n* = 36) – golfers with no history of LBP	– Questionnaire including self-reported LBP in the past 12 months – Body chart	– The weak causal relationship between LBP and hip rotation
McHardy et al., 2007	– 588 Australian amateur golfers (473 men and 115 women)	All Age: 59.1 ± 12.9 years Men Age: 58.7 ± 13.5 years Women Age: 60.8 ± 9.9 years	Men Years of play: 31.8 ± 15.3 Rounds/week: 1.7 ± 1.1 Women Years of play: 21.7 ± 14.6 Rounds/week: 1.8 ± 1.1		– A prospective survey questionnaire	– The observed annual injury rate was 15.8 injuries/100 players (0.4–0.6 injuries/1000 h/person) – Men report higher rate of injuries/100 players than women – The lower back is the most frequently injured part (18.3%) as a result of the golf swing
Saraceni et al., 2017	– 16 recreational male golfers aged ≥ 18 years	Played or practiced golf for an average of ≥4 h/week over the past 2 years	LBP group Years of play: 19 ± 9 Weekly golfing time: 19 ± 16 h No pain group Years of play: 14 ± 6 Weekly golfing time: 23 ± 15 h	LBP group – LBP over 2 years preceding the data collection – 2 or more episodes of LBP – Aggravated by golf – Prevented golfers from playing in 24 months before data collection – Most recent episode occurred in 6 months prior to study	– Clinical examination – motion analysis	– Reduced lead hip internal rotation in the LBP group compared to no pain group during prone passive and standing clinical measures
Joeng et al., 2018	– 363 Korean professional women golfers from Division I, Division II, and Division III	Division I (*n* = 119) Age: 23.5 ± 3.4 years Height: 166.1 ± 5.3 cm Weight: 60.8 ± 5.6 kg Division II (*n* = 121) Age: 22.4 ± 2.9 years Height: 165 ± 5.2 cm Weight: 59.7 ± 6.0 kg Division III (*n* = 123) Age: 21.0 ± 3.2 years Height: 165 ± 5.4 cm Weight: 58.7 ± 8.0 kg	Division I Golf career (years): 12.2 ± 2.6 Tournament games/year: 31 (20 last for 3 days and 11 for 4 days) Hours to complete each round/year: 520 Number of training hours/competition day: 6 Division II and III Golf career (years): 10 ± 2.8 and 7.7 ± 2.7, respectively Tournament games/year: 19 and 16 (2-day tournament games) Hours to complete each round/year: 190 and 160 Number of training hours/competition day: 7 and 7.5		– YISSEM ISS survey	– A total of 510 injuries were recorded – 174 injuries occurred in Division I, 166 in Division II, and 170 in Division III – The most frequent mechanism for injury was the golf swing upon ball impact – The injury risk in female golfers was associated with number of competitions – Lumbar spine/lower back is one of the most common injured part
Dennis et al., 2005	– 44 Australian male fast bowlers compete at club and district levels	Age: 14.7 ± 1.4 years	Bowling days in total (44 bowlers): 1783 (696 match days and 1087 training days) Match/day: 1 Training days/week: 1.5	25% bowlers reported an overuse injury during the season	– Interview with physiotherapist – MRI examination	– An increased risk of injury was observed for those who bowled more than 2.5 days/week or for those with more than 50 deliveries per day – 25% bowlers reported an overuse injury, seven of them sustaining a back injury
Piazza et al., 2009	– 60 former elite women gymnasts from Italian national team – International, national level and Olympic Games competitors	Age: 38 ± 7 years Weight: 54.5 ± 5 kg Height: 1.66 ± 0.44 m	Training intensity: 29.7 ± 11.4 h/week	22 gymnasts with LBP Mean age of symptoms onset: 19 ± 8 years LBP during activity in 71.4% of gymnasts	– Interview – Self-administered questionnaire	– Occurrence of back pain during the sport activity was associated with an early onset of symptoms of LBP after the retire from competitions
Tertti et al., 1990	– 35 competitive gymnasts (18 male and 17 female) – District level gymnast (*n* = 12) – National level gymnasts (*n* = 21) – International level gymnasts (*n* = 2)	Age: 12 ± 2.6 years Weight: 39 ± 9.6 kg Height: 150 ± 13 cm	– Practice time: 12 ± 5.5 h/week – Years of practice: 4.2 ± 2.5		– Questionnaire – Clinical examination – MRI and radiographs	– 11 gymnasts suffer from episodes of LBP during exercises – 8 gymnasts have evidence of back trauma – 3 out of 35 gymnasts have MRI evidence of degenerated discs
Goldstein et al., 1991	– 33 top level female gymnasts (11 pre-elite, 14 elite, national, and 8 Olympic levels) regardless of back pain or injury – Control group: female swimmers	Top gymnasts Pre-elite gymnasts Mean age: 11.8 years Elite gymnasts Mean age: 16.6 years Older national/Olympic gymnasts Average age: 25.7 years Controls The AA and AAA swimmers Average age: 14.6 years Q and national ranked swimmers Average age: 18.6 years	Top gymnasts Pre-elite gymnasts Training h/week: 18.2 in their most current year Elite gymnasts Training h/week: 23.2 in their most current year Older/Olympic gymnasts Training h/week: 22.1 in their most current year Controls The AA and AAA swimmers Training h/week: 11.3 in their most current year Q and national ranked swimmers Training h/week: 16.4 in their most current year	No initial signs of LBP	– MRI examination	– 1 pre-elite, 6 elite and 5 Olympic level gymnasts have spine abnormalities – 15.8% of swimmers (control group) have spine abnormalities – Training volume per week and age are associated with MRI abnormalities – Top female gymnasts are more prone to spine injuries
Reis et al., 2015	– 72 Brazilian jiu-jitsu athletes (36 professional and 36 recreational level athletes)	Median age: 25.5 years	Median training experience: 8 years Training frequency: 2–7 times/week Median duration of each practice: 2 h/day	Athletes without history of LBP before training participation	– Questionnaire – Quebec Back Pain Disability Scale (QBPDS)	– Chronic LBP in 58 of jiu jitsu athletes (32 professional and 26 recreational) – High prevalence of LBP in jiu-jitsu athletes – Professional jiu-jitsu athletes are more prone to developing of chronic LBP
Almeida et al., 2012	– 42 judo athletes (22 male and 20 female)	LBP group Age: 16.7 ± 2.9 years Weight: 60.1 ± 1.34 kg Height: 1.64 ± 0.1 m Control group Age: 16.3 ± 2 years Weight: 61.5 ± 9.9 kg Height: 1.65 ± 0.1 m	LBP group Training experience: 8.9 ± 2.9 years Weekly practice frequency: 5 ± 0.8 Hours of practice/day: 2.1 ± 0.4 Hours of practice/week: 10.1 ± 1.3 Control group Training experience: 8.4 ± 3.1 years Weekly practice frequency: 4.8 ± 0.7 Hours of practice/day: 2.1 ± 0.5 Hours of practice/week 10.5 ± 2.8	Years with low back pain: 1.2 ± 0.5 Number of episodes in previous 12 months: 4.8 ± 8.3 Mean score on visual analog pain scale: 4.1 ± 2.6	– Computed photogrammetry – Pain scale	– Deficits in hip rotation and greater asymmetry between limbs were observed in judo athletes with a history of LBP
Rostami et al., 2015	– 14 professional off-road male cyclists – 24 controls – Cyclists competed at the national and international level during the past 12 months	Off-road cyclists Age: 27.2 ± 4.74 years Height: 172.7 ± 5.5 cm Weight: 71.5 ± 11.9 kg Controls Age: 27.8 ± 5.26 years Height: 179.7 ± 5.66 cm Weight: 80.2 ± 10.5 kg	Off-road cyclists Cycling distance/week: 236.1 ± 116.16 km Road cycling distance/week: 165.0 ± 88.82 km Off-road cycling distance/week: 71.1 ± 40.96 km Controls Cycling distance/week: 299.6 ± 169.13 km Road cycling distance/week: 240.4 ± 161.82 km Off-road cycling distance/week: 58.7 ± 20.50 km	Cyclists with bilateral non-specific LBP for more than 12 months LBP was defined as pain in the area between the 12th rib and the inferior gluteal fold responsible for a limitation in the performing of usual daily activities for more than 1 day during the past 4 weeks	– Ultrasonic measurements – Flexibility assessment – Dynamometry	– Lower thickness of transversus abdominis and cross sectional area of lumbar multifidus spinae in cyclists with LBP compared to controls
Trompeter et al., 2019	– 156 German rowers (104 elite and 52 non-elite / 49 sculls, and 76 sweeps) – 90 males and 65 females – 166 physically active non-rowing controls	Rowers Age: 22.2 ± 5.1 years Height: 183.4 ± 8.3 cm Weight: 78.1 ± 11.5 kg Sex (m/f): 57.1/41.7% Controls Age: 21.2 ± 2.0 years Height: 180.0 ± 8.9 cm Weight: 74.0 ± 10.3 kg Sex (m/f): 74.7/24.1%	Rowers Training volume: 16.3 ± 8.2 h/week Years of rowing training: 9.5 ± 5.4 Controls Training volume: 10.8 ± 5.0 h/week		– Back pain questionnaire – Chronic Pain Grade (CPG)	– High back pain prevalence and severity were observed in rowers than controls, and among scull than sweep rowers – The lower back was the most frequent area for pain occurrence in rowers of all competition levels and also in controls – Age, sex, training volume, kinematics, strength, and ergometer training were associated with higher prevalence of back pain in rowers
Alricsson et al., 2016	– 51 elite Swedish cross-country skiers	Age: 16–19 years Sex (m/f): 30/21	Training hours/week: Pre-season 11.3 ± 2.6 Season 9.0 ± 1.6 Number of competitions/season: 16.5 ± 5.9 Trained 13.8 ± 1.9 (7–16) years	LBP – defined according to European Guidelines (Airaksinen et al., 2006) as pain or discomfort somewhere between the 12th rib and the lower gluteal fold	– Questionnaire about training and competition status – Back pain questionnaire – DeBrunner’s kyphometer – Lumbar locked thoracic rotation test – Modified Thomas test – Prone active straight leg rising	– Participants with greater lordosis than kyphosis suffer from LBP more than those without kyphosis – Sagittal spinal alignment are related to LBP in elite cross-country skiers – The range of motion of the thoracic spine and hips do not affect the prevalence of LBP in elite cross-country skiers
Siewe et al., 2011	– 245 German competitive and elite powerlifters (219 male and 26 female) – 225 powerlifters – winners with 154 medals on the national or international levels	All powerlifters Age: 37.8 ± 14.3 years Weight (off-season): 89.1 ± 18.4 kg Male Weight: 91.9 ± 17.1 kg Female Weight: 65.4 ± 10.2 kg	All powerlifters The maximum average loads lifted: squat 205.8 ± 69.12 kg, bench press 151.6 ± 52.4 kg, and deadlift 214.2 ± 54.8 kg Training experience: 13.6 ± 10.5 years During the competitive season, 88% of subjects worked 3–7 times/week The average workout time: 119.1 ± 39.7 min/day Male The maximum average loads lifted: squat 204.3 ± 69.7 kg, bench press 152.0 ± 52.9 kg and deadlift 213.4 ± 55.2 kg Female The maximum average loads lifted: squat 209.7 ± 64.7 kg, bench press 148.2 ± 48.3 kg, and deadlift 221.6 ± 52.6 kg		– Questionnaire	– Injuries of the lumbar spine in 108 powerlifters – The most frequent diagnoses are sciatica and myogelosis – Gender, exercise weight, workout duration, competition level, and routine endurance training have no impact on injury rates – Using of supporting devices was associated with more lumbar spine problems
Junior et al., 2013	– 191 recreational runners (141 males and 50 females)	All Age: 42.8 ± 10.5 years Height: 171.1 ± 9.4 cm Weight: 72.0 ± 14.0 kg Injured Age: 41.8 ± 10.2 years Height: 172.4 ± 8.8 cm Weight: 73.1 ± 11.8 kg Non-injured Age: 42.9 ± 10.5 years Height: 170.5 ± 9.7 cm Weight: 71.4 ± 14.9 kg	All Distance (km/week): 28.5 (15.0–41.0) Duration (min/session): 60 (50–80) Injured Distance (km/week): 15 (2.5–26.3) Duration (min/session): 50 (15–60) Non-injured Distance (km/week): 30 (18.0–42.5) Duration (min/session): 60 (50–90)	Running related injury was defined as any pain of musculoskeletal origin attributed to running and associated with absence at least one training unit (Bovens et al., 1989; Macera et al., 1989; van Middelkoop et al., 2007, 2008)	– 10-point pain numerical rating scale – Survey	– Running related injuries in lumbar spine in 12 (14%) runners, duration of LBP: 2.4 ± 0.8 weeks, pain intensity: 5.2 ± 2.5
Villavicencio et al., 2006	– 87 triathletes (31 male and 56 female) from Colorado – Elite level (6) – Intermediate level (65) – Beginners (16)	All Age: 36.1 years Women Age: 34.1 years Men Age: 39.8 years Triathletes with acute lumbar pain (*n* = 37) Mean age: 36.3 years Triathletes with subacute lumbar pain (*n* = 8) Mean age: 36.2 years Triathletes with chronic lumbar pain (*n* = 14) Mean age: 36.9 years	All triathletes Number of triathlons: 22.4 Combined training time: 12.5 h/week Swimming, biking, and running accounted for 22.47 and 31% of the training time, respectively Triathletes with acute lumbar pain Training volume (hours/week): 14.9 Number of running races and triathlons: 24.1 Triathletes with subacute lumbar pain Training volume (hours/week): 17.3 Number of running races and triathlons: 24.2 Triathletes with chronic lumbar pain Training volume (hours/week): 15.0 Number of running races and triathlons: 21.4	The incidence of lumbar discogenic back pain was defined according to the duration of symptoms for the most recent pain episode The lifetime incidence of LBP: 67.8% (59 out of 87 triathletes) LBP related to sports injuries: in 48 (81.4%) out of 59 triathletes Triathletes with acute lumbar pain – LBP lasted fewer than 7 days – Triathletes with subacute lumbar pain – Most recent back pain episode lasted from 7 days to 3 months – In 14 out of 59 triathletes, the most recent LBP episode lasted more than 3 months – Number of sports-related injuries: 1.5 – Triathletes with chronic lumbar pain – The most recent LBP episode lasted more than 3 months – 9 triathletes noted sciatica symptoms	– On-line questionnaire	– No significant association between LBP and age, athletic status, or training duration – A strong tendency toward LBP in triathletes with years of experience – Strong association was observed between the number of injuries and the occurrence of LBP
Sekine et al., 2014	– 68 Japanese collegiate rowers (43 men and 25 women) – International competition level (15) – National competition level (49) – Regional competition level (4) – Male rowers mainly participated in sweep oar rowing, while female rowers mainly participated in scull oar rowing	Men Age: 19.4 ± 1.1 years Height: 176.4 ± 5.9 cm Weight: 74.3 ± 8.1 kg Women Age: 19.7 ± 1.1 years Height: 164.2 ± 4.3 cm Weight: 60.0 ± 5.4 kg	Training volume: 11 sessions/week Training duration: 2 h Career duration: 4.8 ± 1.4 years (men) 5.1 ± 2.0 years (women)		– MRI examination – Injury surveillance	– 48.8% of male rowers and 40.0% of female rowers presented disc degeneration – Progression of disc degeneration after 2 years in 5 rowers – LBP during 2 years in 6 rowers; progression of disk degeneration in 4 of them – The prevalence of disc degeneration in lumbar spine among rowers was 45.6%
Roussel et al., 2013	– 40 Belgian pre-professional dancers (2 males, 38 females)	Age: 20.3 ± 2.4 years Height: 1.66.2 ± 0.06 m Weight: 56.43 ± 5.71 kg	Physical activity during classes: 21.5 ± 2.1 h/week Physical activity outside classes: 4.6 ± 1.3 h/week	41% dancers with occurrence of LBP in the last year	– Questionnaires (SF-36, self-established medical) – The Tampa Scale for Kinesiophobia (TSK) – The Visual Analogue Scale (pain severity) – Clinical test battery	– Dancers with a history of LBP presented poorer lumbo-pelvic motor control than those without history of LBP – Pre-professional dancers suffer from LBP frequently – LBP was associated with decreased motor control in dancers
Thoreson et al., 2017b	– 16 elite Swedish Mogul skiers (14 males and 2 females) – 28 age-matched non-athletes (control group)	Mogul skiers Age: 17.6 ± 1.02 years Height: 177.0 ± 6.9 cm Weight: 70.8 ± 10.62 kg Control group Age: 16.4 ± 0.57 years Height: 172 ± 8.56 cm Weight: 67 ± 17.91 kg	Mogul skiers 12 skiers exercised more than 11 h/week 2 skiers exercised 9–11 h/week 2 skiers exercised 6–8 h/week Control group All controls exercised less than 8 h/week 5 controls without exercise experience	Back pain was defined as any present or previous pain in the thoracolumbar back – It was self-assessed – It was graded moderate or severe depends on if daily living and physical activities were affected by pain or not	– MRI examination – Back pain questionnaires – Visual Analogue Scale (the location and type of pain)	– More MRI abnormalities in Mogul skiers compared to the controls (7.25 vs. 3.78) – No difference in LBP lifetime prevalence between skiers and controls – No association between disc degeneration and back pain – Increased risk of developing spinal abnormalities in elite Mogul skiers due to the different high loads than controls
Witwit et al., 2018	– 75 Swedish elite skiers (59 alpine and 16 mogul skiers) – Elite level within the high school competitions – 27 non-athletic first-year high school students (control group)	Elite skiers Age: 18.2 ± 1.13 years Height: 174 ± 8.2 cm Weight: 70.9 ± 9.14 kg Control group Age: 16.4 ± 0.58 years Height: 172 ± 8.57 cm Weight: 67 ± 17.90 kg	Elite skiers 9–11 training hours/week (74%) Control group 2–5 training hours/week (78%)		– MRI examination – Back pain questionnaires	– Higher rate of radiological changes in skiers than controls (more degenerative disc changes in alpine skiers) – No significant difference in lifetime prevalence of back pain between athletes and non-athletes – The occurrence of MRI abnormalities in skiers is not associated with lifetime prevalence of back pain
Angoules et al., 2018	– 46 non-professional Greek classic female ballet dancers	Age: 28.8 ± 5.44 years	Training hours/week: 10.8 ± 6.68 Years of experience: 11.9 ± 4.20		– A self-administered questionnaire	– 31 dancers experienced 3.26 ± 1.7 episodes of mechanical LBP in the previous 12 months and have to refrain from dancing activities (16.9 ± 16.22 days) – 21 dancers received some kind of conservative treatment
Kraft et al., 2009	– 58 elite horseback riders (18 men, 40 women) – Horseback riders competed at a national level, an international level, Olympic Games and/or the World Equestrian Games – Control group: 30 non-riding volunteers (17 men, 13 women)	Age of elite riders: 32.4 years Age of controls: 28.7 years	Training hours/week: 18.5 ± 8.2 Riding experience: 20.1 ± 9.1 years		– Questionnaire – Physical examination – MRI examination – Visual Analogue Scale	– 51 elite horseback riders have a history of LBP – The prevalence of LBP is not different among disciplines – The development of lumbar disk degeneration is not associated with LBP history, discipline, body mass index, and trunk/leg-length ratio – No conclusive MRI evidence to suggest that the cause of LBP in horseback riders lies in structural changes of the spine
Adiele and Morgan, 2018	– 45 competitive swimmers (22 boys and 23 girls) from Zimbabwe	Age: 16.43 ± 2.36 years Height: 168.3 ± 9.8 cm Weight: 55.01 ± 6.14 kg	Years of experience: 3.41 ± 1.52 Training duration: 3.08 ± 1.69 h		– Epidemiological survey	– Musculoskeletal problems and pain in 27 swimmers (16 male and 11 females) – LBP in 17.1% males and 26.2% females
Koyama et al., 2013	– 104 Japanese collegiate gymnasts (70 male and 34 female) – Pre-elite level gymnasts (17) – Elite level gymnasts (71) – National level gymnasts (16). – 2 gymnasts – winners of medals at Olympic and World Championships (4 gold medals)	Age: 19.7 ± 1.0 years	Training experience: 11.8 ± 3.6 years Approximately 4 h of gymnastics training for 6 days/week		– Questionnaire – MRI examination	– More than 1 MRI finding in 49 out of 104 gymnasts – High frequency of lumbar disc degeneration (LDDG) and limbus vertebra is in gymnasts with LBP compared to those without LBP – Only LDDG is a significant variable predictor associated with LBP
						

Regarding the individual sports, these groups of athletes were included: cricket bowlers (1 study), cyclists (5), dancers (2), divers (1), golfers (4), gymnasts (4), horseback riders (1), jiu jitsu athletes (1), judoists (1), powerlifters (1), rowers (2), runners (2), skiers (3), swimmers (2), triathletes (1), weightlifters (1), and wrestlers (1). Studies included athletes from the following countries: Australia (3 studies), Belgium (1), Brazil (1), Colorado (1), England (1), Germany (2), Greece (1), Italy (1), Japan (3), Korea (1), Poland (1), Spain (1), Sweden (5), Switzerland (1), and Zimbabwe (1). Country of origin was not specified in seven studies.

Lumbar spine abnormalities and alterations, low back injuries and pain were diagnosed in many different ways: using imaging systems such as magnetic resonance imaging (MRI), radiographs, CT, Spinal Mouse, motion analysis, ultrasonic measurements, through clinical examination, physical examination, various questionnaires, surveys, body charts, interviews, pain scales, and combinations of the above.

### Overview of Studies Dealing With Back Pain and/or Injury in Athletes of Team Sports

Out of the 17 research articles (Table 2), seven studies (41%) included both sexes, six (35%) were focused on male and four (24%) on female athletes. The number of athletes in respective studies ranged from 29 to 1110 depending on the research methods used (retrospective vs. prospective). Ten out of these studies (56%) dealt with senior players, seven (39%) with junior players and one study (5%) with both age categories. Participants were top athletes (4 studies), elite players (4), non-elite players of different performance levels (5), and players of more than one performance level (5). Top athletes were considered those competing at the international level and taking part in top international competitions (Olympic Games, World Tours, and World Championships). Elite athletes were defined as the members of national teams not competing at the highest level or young athletes who were members of academies for talented youth. Non-elite athletes were from sport clubs, high schools, and colleges.

**TABLE 2:** Training characteristics and identified back problems in athletes of team sports.

**Authors**	**Study design / diagnostics of back pain and/or injury**	**Sport and performance level**	**Subjects**	**Training**	**Back pain and/or injury**	**Main findings**
Fett et al., 2019	– A cross-sectional survey – Questionnaire	– Elite athletes (*n* = 181) of badminton, beach volleyball, handball, tennis, volleyball, and controls – sports students (*n* = 166)	Age of athletes/controls 19.7 ± 4.7/21.2 ± 2 years, body height 181.9 ± 12.3/ 180.1 ± 8,9 cm, body weight 74.5 ± 14.8/74.0 ± 10.3 kg Men/women controls 75:25%/athletes 54:46%	Training volume athletes/controls 17.3 ± 6.6/10.8 ± 5 h/week; Number of competitions 35.3 ± 16.9/0 Playing experience11.6 ± 4.5 years/0	Lifetime, 12-, 3-month, and point prevalence of BP in athletes 85, 75, 58, 38%, in controls 81, 70, 59% (non-significant differences) – Non-significant differences between the sports games and controls – Non-significant differences among sports games – BP location mostly identical for athletes and for controls, and in different sports games – LB the most touched area, followed by the NP and upper back pain – Correlations between BP and training load volume in all groups and pain location	– The relationship between training volume and BP confirmed across all groups
Farahbakhsh et al., 2018	– A cross-sectional survey – Interview and questionnaire with 40 questions related to neck pain (NP) and to LBP	– Young top Iran athletes; 452 male athletes participating in the sport Olympiad 2017 in Tehran Province – Soccer (*n* = 136), volleyball (*n* = 81), basketball (*n* = 52), wrestling (*n* = 69), other athletes (*n* = 39)	Age15.48 ± 1.81 (other sports) to 16.1 ± 0.93 (soccer); Body height from 165.02 ± 16.55 (other sports) to 176.71 ± 9.09 (basketball); body weight from 57.16 ± 18.10 (other sports) to 72.94 ± 15.44 (wrestling)	Mean training hours/week 9.71 ± 5.68 (soccer), 11.69 ± 6.94 (volleyball), 11.29 ± 4.64 (wrestling), 11.57 ± 8.22 (basketball), 11.37 ± 18.85 (other sports) Mean experience in sport 5.1 ± 2.53 year (soccer), 2.69 ± 1.48 years (volleyball), 3.51 ± 2.91 years (wrestling), 3.77 ± 1.62 years (basketball), 4.43 ± 2.67 years (other sports)	– Basketball players the highest life-time prevalence of NP (57.69%) – Volleyball players the highest life-time prevalence of LBP (67.90%) – Wrestlers the lowest life-time prevalence of NP and LBP (18.84, 10.14%) – Basketball players the highest risk of NP in all time points (1.54–7.25) – Basketball players the highest risk of LBP at most time points – Wrestlers the lowest life-time risk of LBP (0.064–0.36)	– A high prevalence of neck pain and LBP in young athletes – The highest risk of NP and LBP in basketball players and the lowest in wrestlers
Leppänen et al., 2015	– Retrospective analysis as part of 3-year follow-up study; 12-month period – A detailed questionnaire, based on a previous study of sports injuries	– 18 of 20 basketball and floorball teams of the Tampere region; 207 basketball players (boys *n* = 101, girls *n* = 106); 194 floorball players (boys *n* = 112, girls *n* = 82)	– Floorball players/ basketball players: age 16.6 ± 1.4/ 14.8 ± 1.5 years, BMI (21.9 ± 2.2/ 21.4 ± 3.0 kg/m^2^, playing experience 7.6 ± 3.0/6.9 ± 2.9 years	Playing experience of basketball players: boys/girls 8.1 ± 3.1/ 6.3 ± 2.6 years, floorball players 7.6 ± 3.0 vs. 6.9 ± 2.9 years. Training session/week of boys/girls 4.3 ± 1.5/3.8 ± 1.2; training session/hour 10.2 ± 3.9/9.0 ± 3.0; game/season 35.2 ± 15.3/37.1 ± 16.5	– Basketball: boys 44 (45%) overuse injuries, girls 53 (55%), in total 97, 0.47 overuse injuries per athlete/year; most of the overuse injuries at the lower extremities (64 cases, 66%), knee (44 cases, 45%), LBP (27 cases, 13%) without differences between boys and girls; average time loss from training participation 26 ± 50, median 7 days – Floorball: 72 (37%) of players at least one overuse injury (51 boys and 21 girls); the most common injuries location the lower back/pelvis (36, 39%), the second the knee (32, 34%); boys significantly LB and knee overuse injuries than girls; average time loss 16 ± 37 (median 5) days; the ratio of overuse injuries per athlete/year 0.47	– The prevalence of overuse injuries is rather high already in youth basketball and floorball categories
Anza et al., 2013	– 4 months prospective study – Nordic musculoskeletal questionnaire (NMQ) on prevalence of musculoskeletal symptoms	Volleyball, sport clubs	Boys (*n* = 19) (185.6 ± 6.72 cm), Girls (*n* = 19) (171.8 ± 9.28 cm), 14–17 years. Inclusion criterium: at least 1 year of regular training	Weekly training hours: boys 10.78 ± 1.76 h, girls 14.11 ± 2.08 h	– The most symptoms in 4 months found in the back (56.5%), shoulder (52.2%), hips/thighs (52.2%), and knees (52.2%) – Small changes of the prevalence regarding musculoskeletal symptoms and pain in boys/girls in 4 months (LBP in the last 7 days 44.4/43%, in the last 12 months 55.0/56.5; players impaired of sport activities 17.4/11.1%, professional treatment of LBP 11.1/13% of players)	– Training load was not a factor of musculoskeletal symptoms and pain
Augustsson et al., 2006	– Retrospective study – Questionnaire designed by the first author	Volleyball, elite Swedish division players	– 225 volleyball players of 10 men’s and 9 women’s teams – Inclusion criterium: players of regular team line-up including substitutes – Age: men 25 ± 4 years; women 24 ± 4 years – 47% of men and 53% of women returned the questionnaire	– The total time of training and matches 31,972 h – The number of training hours per week for men 9.9 ± 4 h and 9.4 ± 7 h for women – Match playing time 7 h/week for men and 0.6 h/week for women	– Most of the injuries in the ankle (23%), followed by knee (18%) and back (15%) – Most injuries (62%) of minor severity – Most injuries during training (47%), 41% a gradual onset – 96% of players performed prevention training, mostly without supervision (58%)	– Without distinguishing between acute and overuse injuries, 45% of them occurred during blocking, 30% during spiking
Bere et al., 2015	– Prospective registration of injuries during all major FIVB tournaments – Medical reports	– Junior and senior, male and female volleyball players at major FIVB events (23 senior and 9 junior events) – 4-year data obtained through the FIVB Injury Surveillance System (ISS)	– 32 events, 2640 of 2710 report forms from team medical staff, (response rate of 97.4%) – 440 injuries reported (62.5% during match play, 37.5% during training)		– In all age and sex groups, the ankle the most injured body part (25.9%), followed by knee (15.2%), finger/thumb (10.7%), and lumbar/lower back (8.9%); similar distribution between matches and training – The most common injury: joint sprains (32.5%, *n* = 143) and muscle strains (14.1%, *n* = 62); most muscle strains in the lower back (*n* = 19) – Senior players a higher risk of injury than juniors – No difference between males and females – 23.0% of all injuries contact injuries, 20.7% overuse injuries, 17.3% non-contact injury	– Most injuries related to sport-specific movement patterns (repetitive jumping and landing, spiking, blocking and serving)
Bahr and Reeser, 2003	– Cohort study – Retrospective injury reports with prospective registration of injuries over a 7.5-weeks – Interview/report of tournament medical staff	Beach volleyball, players at the FIVB World Championships 2001	Professional male and female top beach volleyball players (178 out of 188 participants) from 30 different countries	– 7.5 weeks of training and competitions before World Championships (6 female tournaments and 8 male tournaments on the World Tour, and other national or regional tournaments) – Men: 2410 match hours, 1071 warm up and 6295 h of total training – Women:1832 match hours, 814 warm up and 6493 total training hours	– 54 of acute injuries recorded in the retrospective survey, and 25 in the prospective study – 67 players (38%) 79 overuse injuries in the retrospective survey with medical attention – Most common overuse injuries LBP (15%), knee pain (12%), and shoulder problems (10%) – Injuries in relation to body region in the prospective study similar to that of retrospective study anyway much lower	– Low back (LB) and shoulder overuse problems attributed to frequent spiking and jump serving – LB problems in players depend on the sand quality and its depth, heavy wet balls when raining (larger loads for the shoulder and lumbar spine during spiking and serving)
Külling et al., 2014	– Cross sectional study – Structured interview; MRI examination	– Beach volleyball; – Grand Slam Tournaments – Fully professional male players with or without back pain; a world ranking in the top 100	Age of players (*n* = 29) 28 years (19–39 years), body height 187 cm (179–205 cm), body weight 85 kg (63–100 kg), BMI 22.6 kg/m^2^ (19.7–25.6 kg/m^2^)	– The average duration of professional beach volleyball activity 9.8 years (2–20 years) – The average training hours/week 18.5 (9–35 h)	– 86% of players LBP during their career, in the last 4 weeks 35% – Averaged pain at a visual analog scale (VAS) 3 points (0–8) – 23 out of 29 players (79%) at least 1 degenerated disc of Pfirrmann grad >3; the most affected spinal levels L4–5 in 14 (48%) and L5–S1 in 15 players (52%); both levels involved in 5 players (17%) – 6 (21%) players a spondylolysis grade 4 according to the Hollenburg classification; spondylolisthesis in 2 players – Non-significant correlation between LBP and MRI abnormalities	– High prevalence of disc degeneration and spondylolysis in the lumbar spine in professional beach volleyball players – No correlations between LBP and MRI abnormalities in the study subjects
Bacon and Mauger, 2017	– Prospective cohort study – Volume and intensity variables derived from GPS (StatSports, Viper Pod, NI) – Injuries collected by a qualified physiotherapist	– Professional youth soccer players (*n* = 41), Barclays U21/U18 Premier Leagues – Data collection 40 weeks of the competitive season for training sessions and matches	– Season 2012/2013 (*n* = 18), age 18.7 ± 1.2 years, body height 175.2 ± 4.5 cm, body weight 72.4 ± 3.1 kg – Season 2013/2014 (*n* = 23), age 17.0 ± 1.1 years, body height 181.3 ± 6.1 cm, body weight 74.9 ± 8.7 kg	– Players either with a signed youth scholarship contract or a professional contract with the club – 6 training sessions per week	– Of the 190 injuries 5.46 IIR, 51.76% (*n* = 44,5) non-contact – Total distance significantly predict overuse injury rates (*F*_1,39_ = 6.482, *p* = 0.015), high-speed running meters not (*F*_1,39_ = 1.003, *p* = 0.323) – Back injuries very low rate (0.25/1000 h, 2.4%)	– The incidence of overuse injury impact distance covered in training and matches, not the high-speed running meters – Cumulative approach to training load has value when assessing players injury risk
Pasanen et al., 2008	– 6 months prospective study – Structured questionnaire registered by player and verified personally by a physician	Floorball, licensed female players from Finnish top leagues (*n* = 374) – Players’ level: elite league (*n* = 164), first division (*n* = 183), second division (*n* = 27), – Field position: goalkeeper *n* = 41, defender *n* = 120, forward *n* = 213	Age 24.2 ± 4.8 years, body height 166.2 2 ± 5.2 cm, body weight 61.6 2 ± 6.5 kg	– 122 practice hours and 5.9 match hours/player in average – Elite league 133 practice hours (95% CI 124.9–141.5 h), first division 111 h (95% CI 103.9–117.9 h), second division 124 h (95% CI 103.9–143.7) – Intensive game load average during the season: elite league players 7.5 h (95% CI 6.8–8.1 h), first-division players 4.7 (95% CI 4.2–5.1 h), second-division players 4.7 h (95% CI 3.8–5.6 h)	– 73% of the injuries traumatic and 27% from overuse – Most of the traumatic injuries in ankle and knee (29/28%) – Most of the overuse injuries in knee, calf/shin (22%) and back (14%) – The injury rate per practice hours 1.8/1000 – Overall injury rate/1000 game hours 40.3, in the elite league 34.3, in the first division 48.0 and in the second division 47.7 – 56% of injuries treated at clinics, 44% by the players themselves, 6% needed hospital admission	– No clear reason of a high incidence of overuse injuries and non-contact injuries – Possible factors – type of floor and shoes, lack of proprioception and conditioning training
Haydt et al., 2012	– Cross sectional study – Pen and paper survey questionnaire on incidence of LBP	– Female NCAA Division III field hockey players (FH); season 2008 (*n* = 90); age-matched controls from Misericordia University (*n* = 98), without participation in FH – Inclusion criteria: females 18–24 years with a self-reported LBP lasting more than 24 h not associated with menstruation	Age FH players 19.28 ± 1.19 years; range 18–22 years; controls 19.89 ± 1.45 years; range 18–24 years	– Not mentioned although they were surveyed	– Similarity in incidence of LBP in the FL players and controls (56% in FL players, 55% in controls) – Onset age of LBP for controls and the FH was 16.45 ± 2.12 and 16.23 ± 1.80 years – Duration of symptoms was less than 3 weeks in controls (85%) and FH group (82%) – Episode of LBP of 1 month or longer was 15% in controls and 14% in FH – Similarities in pain distal to the buttock in both groups	– Non-significant differences in the incidence of LBP and pain characteristics between the groups – In both groups a high incidence of LBP (>50%) with an onset of LBP at a mean age of approximately 16 years
Jacobson and Tegner, 2007	– Prospective cohort study – Injuries registered by trainer/coach, in national teams by physiotherapist using standardized protocols (Ekstrand, 1982) – Injured players interviewed by the first author by telephone	Swedish female elite soccer players in premiere league, divided in national (*n* = 51) and no-national players (*n* = 144)	– 195 of 269 players (72%) – Age 23 ± 4 years, body height 168 ± 5 cm, body weight 62 ± 7 kg, BMI 22 ± 2	– On the average 140 ± 48 (24–238) practice sessions and 35 ± 12 (2–48) matches – Players of national teams – 52 practice sessions and 23 matches more – Total football hours national/non-national players 321 ± 134/ 257 ± 78 h	– Traumatic injuries 163/237 (69%, 3.3/1000 h of football) – Overuse injuries 74/237 (1.3/1000 h of soccer) – Overuse injuries mostly during pre- and at the beginning of spring season – The highest incidence in the knee, the lower leg, and the back (39/18/16%; 0.6, 0.3, and 0.2/1000 h of soccer, respectively	– Back the third of most common body location of acute and overuse injuries – National team players and players in the three highest ranked teams although exposed to more playing hours during the year not different in injury incidence as non-national players – Injured players higher practice/game ratio than non-injured players – High amount of re-injuries (>50%)
Tunås et al., 2015	– Prospective cohort study – Standardized Nordic musculoskeletal questionnaire (NMQ)	– Female elite soccer players (2009–2011; 3 years) (*n* = 267) – Female elite handball players (2007–2011; 5 years) (*n* = 173) – Female controls (2012) (*n* = 400) practicing no more than 5 times per week	Soccer/handball players/controls: Mean age 22.4 ± 4/22.3 ± 3/ 25.6 ± 4 years, Body height 167.7 ± 5/173.1 ± 6/ 167.9 ± 7 cm, Body weight 62.6 ± 7/69.0 ± 7/ 66.1 ± 14 kg, Playing at elite level 3.1 ± 4/3.2 ± 4 years	Training volume of players: – Soccer: less than 400 h/year 5.2%; 400–699 h/year 74.5%; more than 700 h/year 20.3% of players; – Handball less than 400 h/year 5.8%; 400–699 h/year 73.4%; more than 700 h/year 20.8% of players	– No differences in the prevalence of LBP among the groups – More than 60% players of the groups experienced LBP ever, more than 50% during previous 12 months and 24–31% in the previous week – Only 3.2% of handball, 3.6% of football players and 4.9% controls had no experience with LBP – More than 70% of football and handball players with LBP stated as the source the overuse, less than 10% traumatic injury	– Non-significant differences in the prevalence of LBP among soccer players, handball players and the control group – High frequency of LBP among examined players; as the main source of LBP stated overuse
Haag et al., 2016	– Cross-sectional survey based on a retrospective data – An iPad-compatible questionnaire; a multiple-logistic-regression model – Nomogram based on the ORs of each parameter	841 male, 269 female soccer players from Bavarian soccer clubs (74 different clubs, 75.0% from 1480 athletes)	Age of boys 15.96 ± 1.52 years, girls 14.38 ± 1.67 years, body height of boys 174.96 ± 9.03 cm, girls 163.82 ± 6.64 cm; body weight of boys 64.59 ± 11.74 kg; girls 52.64 ± 9.11 kg	Mean soccer training in boys 8976 ± 2.94 years (7–11 years) and in girls 3986 ± 3.5 years (3–8 years)	– The greatest impact on prevalence of back pain (BP) the age, followed by sex and playing surface (U19/U17/U15, 1.84/1.66/1.11) – Female players a greater risk (OR = 1.48) for BP than the boys; higher BMI linked to lower amount of BP (OR = 0.97); longer training history a negative effect on BP (OR = 1.04); more than 6 or less than 3 training h/week significant effects on the prevalence of BP (OR = 0.81); natural grass the highest negative influence (OR = 1.56); goalkeepers specific injury prevalence and incidence	Among all factors, longer training history and very high or low training load have an impact on the prevalence of BP
Bowen et al., 2017	– 2 year prospective study – Workload quantified using GPS (Viper V.2, StatSports, Ireland), data from all on-pitch training sessions and matches – Injury report classified by doctors and physiotherapists	Elite youth soccer players (*n* = 32), category academy U18, U21 Premier League 2013–2014 and 2014–2015 seasons	Age 17.3 ± 0.9 years, body height 180.0 ± 7.3 cm, body weight: 74.1 ± 7.0 kg	– Training on a full-time basis – Competition: 63% of participants in both seasons and 38% participants in one season	– Very high number of accelerations (≥9254) over 3 weeks associated with the highest relative risk (RR) (RR = 3.84) and non-contact injury risk (RR = 5.11) – Non-contact injury risk higher by combination of high acute high-speed distance (HSD) with low chronic HSD (RR = 2.55), but not with high chronic HSD (RR = 0.47) – Most common non-contact injuries in ankle/foot (2.1/1000 h), hip/groin (1.3/1000 h), knee (1.1/1000 h), and abdomen/back (0.4/1000 h)	– High accumulated and acute workloads are associated with a greater injury risk – The injury risk is greatest by very high number of accelerations accumulated over 3 weeks – Progressive increases of chronic workload develops the players’ tolerance to higher acute loads and reduces injury risk
Aasheim et al., 2018	– Prospective cohort study (7 months) – OSTRC-O questionnaire distributed by email every second Sunday, software SurveyXact V.8.2, Rambøll Management Consulting, Oslo, Norway	– Male handball players aged 16–18 years (*n* = 145) from South-Eastern Norway during the 2016–2017 season – Complete data from 48% of players	– 61% of players 17 years old, 33% 18 years old and 6% 16 years old; body height 184 ± 7 cm (172–198 cm), body weight 80 ± 11 kg (60–115 kg) – 55% of players participated in two different teams (senior/junior), 28% in one team, 17% in three teams, 10% players in junior national team	– Handball training: 9 ± 2 years (4–14 years). – The median amount of training during the study: 14 h/week, approximately 6 h handball, 4.5 h strength, 3 h alternative training and 30 min handball match play	– Average prevalence of all overuse injuries 39% (95% CI 29–49) – Average prevalence of all substantial overuse injury problems 15% (95% CI 13–17) – Highest average prevalence in shoulder (38% of players), knee (36%), and low back (26%); overuse injuries in each anatomical area after 7 months about 20% higher than at baseline	– The prevalence of overuse injuries is high, especially in the shoulder, knee, and low back – Tendency of worsening during the season
Hoskins et al., 2009	– A cross-sectional study – The questionnaire using Quadruple Visual Analogue Scale of the McGill Pain Questionnaire (short-form) with LBP questions adapted from an Australian LBP epidemiological study	– 271 elite players, 360 semi-elite players, 148 non-athletic controls – At the elite/semi-elite level Australian rugby league players (*n* = 93/52), rugby union players (*n* = 19/139), Australian-Rules players (*n* = 112/90) and soccer players (*n* = 47/70)	Age of elite/semi-elite/non-athletic controls 23.3 ± 4 years, 23.8 ± 4 years, and 23.9 ± 4.5 years	–	– Almost linear trend of increasing LBP severity from non-athletes, to the semi-elite and elite athletes – Levels of the sensory, affective and total pain score significantly higher in elite athletes – Elite athletes approximately two times more experienced discomforting or greater LBP, and less likely no LBP as other groups; semi-elite athletes less likely experienced discomforting or greater LBP; and non-athletes more likely not experienced LBP (χ^2^ = 18.67, *p* < 0.001) – The age of first episode of LBP – between groups non-significant differences – Elite athletes 2–4 times attributed sporting activity as a cause of current LBP	– Elite players had significantly higher levels and more frequent LBP – LBP in elite players was more attributed to sporting activity when compared to semi-elites and non-athletes – The difference of onset of the first time LBP non-significant between groups – Reported etiology between groups is different
						

Regarding the team sports category, the following groups of athletes were included: soccer players (7 studies), volleyball players (5), beach volleyball and handball players (3), field hockey, floorball, handball and basketball players (2), and the players of different types of rugby (1). Studies included were from the following countries: Norway (4 studies), Germany (3), United Kingdom, Sweden, and Finland (2), Switzerland, United States, Australia, Iran, and Portugal (1). Players included were from the same countries except of three studies which were conducted at major international events such as World Championships or World Tours.

This review includes two main types of studies: prospective cohort studies and retrospective studies. Low back injuries and pain or lumbar spine abnormalities were diagnosed in the following ways: using imaging systems (MRI), through physical examination by medical staff or physiotherapists, questionnaires, interviews, and their various combinations.

### The Association of Workload With the Occurrence of Back Problems in Athletes of Individual and Team Sports

There is no conclusive evidence to date to demonstrate that the cause of low back pain in athletes of individual sports lies in undue abnormalities of the spine. Only one study (Koyama et al., 2013) confirmed lumbar disc degeneration as a statistically significant variable accounting for low back pain. However, no direct association between the training load and back injuries or abnormalities in the spine was confirmed. Nonetheless, there were trends toward an increased risk of injuries with increasing levels of performance and high physical loads.

Based on the MRI studies (Tertti et al., 1990; Baranto et al., 2006; Kaneoka et al., 2007; Kraft et al., 2009; Koyama et al., 2013; Sekine et al., 2014; Thoreson et al., 2017b; Witwit et al., 2018), the most common abnormalities in athletes of individual sports were as follows: reduced disc signal, degenerated discs at various disc levels, ring apophyses injury, disc bulging, Schmorl’s nodes causing severe back pain, abnormal configuration of the vertebra, and alterations in spinal curvatures.

In team sport games, the prevalence of overuse low back injuries accompanied by low back pain is rather high. However, the research findings are inconsistent. The level of prevalence depends on the type of research design used. While in prospective studies the prevalence of low back pain was evident in 9–20% of the cases (Bahr and Reeser, 2003; Jacobson and Tegner, 2007; Pasanen et al., 2008; Bere et al., 2015; Aasheim et al., 2018) or 2.25−0.4/1000 training and match hours (Bacon and Mauger, 2017; Bowen et al., 2017), in the retrospective studies it occurred in 15–97% of the cases depending on time to occurrence of back problems (point, 1-week, 1-, 3-, 12-month, life time prevalence) (Hoskins et al., 2009; Haydt et al., 2012; Külling et al., 2014; Leppänen et al., 2015; Tunås et al., 2015; Haag et al., 2016; Farahbakhsh et al., 2018; Fett et al., 2019).

The highest prevalence of low back pain was identified in field hockey, floorball, rugby, and beach volleyball (Nosco et al., 1999; Pasanen et al., 2008; Hoskins et al., 2009; Haydt et al., 2012; Külling et al., 2014; Fett et al., 2019). Among pain caused by overuse, low back pain was the most often reported pain with an incidence of 20–86% depending on the age category, sex, performance level, and the time of occurrence. In soccer, volleyball, and handball, low back pain was the third most common pain caused by overuse (Augustsson et al., 2006; Anza et al., 2013; Bere et al., 2015; Tunås et al., 2015; Bowen et al., 2017; Aasheim et al., 2018). In soccer, pain caused by overuse of groin and hip with a rate of 38–57% was the most common, followed by low back pain caused by overuse, whereas in volleyball and handball overuse injuries of shoulder and knee at a rate of 46–57% and 12–59%, respectively, were the most common, followed by low back pain.

The risk factors also include training and match load, which is characterized by the specific movement pattern of sport games, such as unilateral load, repetitive static flexion, hyperextension combined with rotation of the spine, jumps, impacts etc. (Snellman et al., 2001; Pasanen et al., 2008; Bere et al., 2015; Fett et al., 2019). However, the evidence is not unambiguous. The findings are mostly based on comparisons of athletes and non-athletes, and players with other athletes, top, elite, and non-elite players (Bahr and Reeser, 2003; Jacobson and Tegner, 2007; Hoskins et al., 2009; Anza et al., 2013; Bere et al., 2015; Haag et al., 2016; Aasheim et al., 2018).

In recent years, more attention has been directed toward objective quantification of training and match load, especially using the ACWR, and the examination of its relationship with injury risk (Griffin et al., 2020). Although 15 studies dealt with this issue in team sport games, only 2 of them can be included in this review (Bacon and Mauger, 2017; Bowen et al., 2017). In other studies, there was no distinction between the type and site of injuries.

Overall, the literature supports the association of training load with the occurrence of back problems in athletes of individual and team sports. A review by Moradi et al. (2015) identified many potential general and sport-specific risk factors for back pain in athletes, however, the evidence exists only for previous low back pain, decreased lumbar extension or flexion, and high body weight. Differences in the association between potential risk factors and back pain may be ascribed to the type of sport, level of competition, and training characteristics (Moradi et al., 2015). Evidently, properly designed training programs might not affect postural and core stability or increase the risk of injuries. For instance, lower lumbar lordosis and thoracic kyphosis, as well as anterior pelvic tilt while standing were identified in Latin American style professional dancers (Muyor et al., 2017). Though specific postures and movements in dance modify their spinal curvatures, it does not alter the spinal morphology in standing when compared to non-dancers. Further, Belavý et al. (2017) demonstrated that chronic running is associated with better intervertebral discs (IVD) composition and IVD hypertrophy. Generally, pain perception and processing is different in athletes as compared to normally active controls (Tesarz et al., 2012). This suggests a compensatory response of the endogenous antinociceptive system to the noxious input during exhaustive endurance training (Tesarz et al., 2013). More specifically, an overstressing of the endogenous pain inhibitory pathways may lead to exhaustion over time (Tesarz et al., 2013). This may result in disinhibition of pain processing when the acute pain is transmitted in the chronic pain and spatial pain spreading (Tesarz et al., 2013). On the contrary, the endogenous pain inhibitory pathways may be protected from chronic overstressing over time by a shift in the activation threshold, which may increase the efficiency of pain inhibition (Tesarz et al., 2013). Specific changes in pain thresholds and higher tolernce to pain at rest in athletes (Tesarz et al., 2012) has to be also taken into account when interpreting self-reported back pain and its relationship with physiological loading of the spine in athletes.

Actually, training is a protective factor against injury. As shown, high chronic workloads decrease the injury risk (Gabbett and Domrow, 2005). A lower risk of sustaining a subsequent injury was reported in athletes of team sports when they trained over 18 weeks before their initial injury (Gabbett and Domrow, 2005). A decreased risk of injury is also associated with well-developed physical qualities (Quarrie et al., 2001; Gabbett and Domrow, 2005; Gabbett et al., 2012; Gastin et al., 2015). Thus, progressively increased high training loads not only improve physical fitness of players but may also protect against injury (Gabbett, 2016). For instance, heavier rugby players with faster speed generate greater impact forces which may increase the recurrent rates of contact injuries (Gabbett et al., 2012). However, greater intermittent running speed at high intensity as well as muscle strength and power may decrease the risk of injuries in these players (Gabbett et al., 2012). This indicates that not only overtraining but also undertraining may increase the injury risk (Lyman et al., 2001; Orchard et al., 2009; Cross et al., 2016).

So far, the relationship between the ACWR and back pain/or injury in athletes remains unclear. This is mainly due to the limited evidence and varying methodological quality of particular studies. Nonetheless, it is most likely that sudden spikes in acute training loads may have detrimental effects, whereas high chronic loads and adequate strength and endurance of core muscles could provide protective effects on increasing the occurrence of back pain and/or injury. A structured back-strengthening program has been recommended for reduction of back pain experienced by athletes (Trainor and Wiesel, 2002). In practice, however, functional strength training for back pain prevention can be similar to programs for back pain rehabilitation (Wirth et al., 2017). Most exercises have not been tested for their effectiveness and compared with the load used for strength training. Neither are core strength exercises more effective than traditional resistance exercises (Smith et al., 2014) in individuals with back pain (Macedo et al., 2016; Saragiotto et al., 2016). Adaptations in voluntary activation of trunk muscles to core stability exercises have provided a basis for exercise guidelines (Wirth et al., 2017). However, adaptations of morphological structures that are essential for the core stability have not been sufficiently addressed in research studies. Therefore, the guidelines used for rehabilitation of back pain are insufficient for strength training in athletes (Wirth et al., 2017). Currently, there is no evidence-based exercise program for athletes with back pain and/or injury. Most studies investigated only a part with a narrow group of athletes who usually performed different types of exercise (Daniels et al., 2020). Recent evidence suggests that poor load management is a main injury risk factor (Soligard et al., 2016). Recently, the IOC provided the consensus statement that include guidelines for monitoring of training, competition and psychological load, prescription of training and competition load, athlete well-being and injury (Soligard et al., 2016).

### Gaps in Current Studies Investigating the Workload in Relation to Back Problems in Athletes and Proposals for Future Research

One of the former studies reported that the incidence of injury is increased when the duration, intensity, and load of training sessions and matches in rugby league is increased (Gabbett, 2004a; Gabbett and Domrow, 2007). There is a U-shaped relationship for 4-week cumulative loads with an increase in injury risk with higher loads (Cross et al., 2016). The risk of injury in professional rugby union players increases if they have high 1-week cumulative loads or large week-to-week changes in the training load (Cross et al., 2016). Further, higher risk of injury is also associated with a large increase in acute workload in elite cricket fast bowlers (Hulin et al., 2014). A sharp increases in running workload, the ACWR > 2.0 for either total distance or a high-speed distance, increases the likelihood of injury in elite footballers in both a given and the subsequent week (Murray et al., 2017). Controlling for training load in a given week may decrease the odds of injury in the subsequent week. Windt et al. (2017) demonstrated that increasing participation in pre-season training may decrease the risk of in-season injury in elite rugby league players. Though increased amounts of high-velocity running are associated with higher risk of soft-tissue injury of the lower body, distances covered at moderate and low speeds are protective against this injury (Gabbett and Ullah, 2012). Decreasing the training load during an early-competition phase can reduce the odds of injury without compromising the agility performance in athletes of collision sports (Gabbett and Domrow, 2007). Likewise, reductions in pre-season training loads decrease training injury rates and at the same time increase the maximal aerobic power in rugby league players (Gabbett, 2004b). It seems that high chronic training loads may decrease the risk of injuries in athletes. For instance, elite rugby league players are more resistant to injury when train at a high than a low chronic workload with ACWRs 0.85–1.35, whereas they are less resistant to injury when ACWRs is ∼1.5 and they are subjected to“spikes” in acute workload (Hulin et al., 2016b). Very high and high chronic workloads have protective effects against match injury following shorter recovery periods between matches (Hulin et al., 2016a). Alternatively, a high aerobic capacity and playing experience protect injury in elite Gaelic football players against rapid changes in workload and high ACWRs > 2.0 (Malone et al., 2017a). Players better tolerated increased distances and exposures to maximal velocity when train at higher than at lower chronic loads (Malone et al., 2017b). Over- and under-exposure them to maximal velocity increased the injury risk (Malone et al., 2017b). Accordingly, high chronic workloads, combined with reductions in acute workloads before competition, decrease the risk of injury but might also improve sporting performance (Gabbett, 2016). In general, ACWRs ≥ 1.5 (i.e., greater acute than chronic training load) are associated with a higher injury risk than ACWRs in the range from 0.8 to 1.3. While most studies showed the association of Acute:Chronic markers with risk of injuries, its application in predictions of injuries is questioned (Fanchini et al., 2018; McCall et al., 2018). Rather, it can identify workloads that athletes should use to make a decision when risk of injury is increased or decreased (Hulin and Gabbett, 2019). Therefore, the ACWR should not be viewed in isolation.

Contrary to these studies, the suggested association between training loads and back pain and/or injury is based on case studies, expert opinions, and unpublished data, whereas some other findings are controversial. Analysis of the literature identified several limitations in relation to workloads and back problems in athletes. First, the studies that use the same methods are limited. Second, injury identification is often based only on players’ self-assessment. Third, the exposure hours are calculated as average values for training and playing, and in most cases are based on data reported by individuals. Additionally, prevalence of back pain and/or injury in some sports can be affected by sample size. Finally, numbers of injury definitions and time occurrence categories make a comparison among studies more difficult. Further studies using a wider methodological approach are necessary to deepen the knowledge and understanding the association between the training load, especially ACWR and back pain and/or injury in athletes of individual and team sports.

Because questionnaires are mainly used for identification of back pain, more objective methods evaluating spine curvature and flexibility (Muyor et al., 2017), strength, power and endurance of core muscles (Zemková et al., 2016a, 2017, 2019a,b; Zemková and Jeleň, 2019) and hamstrings (Zemková et al., 2015), as well as postural and core stability after perturbations (Zemková et al., 2016b) should be applied. In addition to sport-specific testing methods, basic physical fitness tests can be used (Zemková and Hamar, 2018). Athletes undergoing the high-level of training demonstrated similar deconditioning of the lumbar extensor muscles and used similar strategies to maintain spinal stability after unexpected perturbations to ensure the spine from pain and damage as compared to non-athletes with low back pain (Catalá et al., 2018). Assessment of training interventions for strengthening the core muscles is also necessary, not only in adult athletes with a predisposition to back pain and/or injury but also in adolescents (Zemková and Hamar, 2018). There is increasing evidence that an experience of back pain in 14-year-old individuals may be associated with pain in adulthood (Coenen et al., 2017). Repetitive flexion and extension loadings of young Functional Spinal Units causes MRI and histological changes in the growth zones and endplates that are most likely first signs of fatigue and an explanation for the spine injuries in adolescent athletes (Thoreson et al., 2017a). In spite of the fact that back pain and/or injury is one of the most prevalent diagnosis in athletes, the mechanism of back problems is poorly understood so far (Ball et al., 2019). Research studies conducted on athletes with back pain highlight physiological and biomechanical mechanisms as influential, whereas psychological factors are often neglected. Heidari et al. (2017) emphasizes the importance of mainly stress-related factors in prevention and rehabilitation of back pain. Therefore, more longitudinal studies are required in comparison with frequently conducted short-term experiments in order to investigate long-term changes in strength of core muscles and neuromuscular control of spine stability in athletes.

Collectivelly, the association between the ACWR and injury in a variety of sports has been well established. However, our review highlights the paucity of research studies directly evaluating the association between the ACWR and back pain and/or injury across a larger range of sports. The inconsistency in the methodology of calculating weekly training loads and missing specification of injuries are main limiting factors that restricted comparison between findings. To reduce the variability between studies, researchers need to clearly define the ACWR methodology and specify back problems. The acute and chronic time periods used for assessment of the ACWR may depend on the specific nature of particular sports. Future investigations would benefit from more information on the fundamentals of competition and training in particular sports, physical fitness attributes of athletes, and other back pain and/or injury risk factors.

## Conclusion

Although the relationship exists between the training load and the occurrence of back problems in athletes of individual and team sports, this is not specifically related to ACWR. In comparison with biomechanical, physiological or stress-related psychological factors, sport-specific patterns underlying high prevalence rates are not fully elucidated. The fatigue of trunk muscles induced by excessive loading of the spine is one of the sources of back problems in athletes. Practitioners can address this issue by load management via the use of the ACWR. However, they should be aware that the acute and chronic time window may vary according to specific structure of individual and team sports and their training and competition schedules. Though this may provide important feedback on athlete’s workload, the questionable is the sensitivity and specificity of the ACWR to predict back problems. This is mainly due to the limited evidence on applications of this systems-model approach for identification of risk of back pain and back injuries. Future research should focus on investigation of ACWR validity in combination with multifaceted monitoring systems in prevention of back problems in athletes of both individual and team sports. Nonetheless, the ACWR can be used as an effective tool for monitoring the athlete’s responses to training thereby enable practitioners to design workloads that decrease the occurrence of back problems. It seems that high chronic workloads and adequate muscle strength and endurance have a protective effect on increasing the occurrence of sport-related injuries.

## Data Availability Statement

The raw data supporting the conclusions of this article will be made available by the authors, without undue reservation, to any qualified researcher.

## Author Contributions

All authors listed have made a substantial, direct and intellectual contribution to the work, and approved it for publication.

## Conflict of Interest

The authors declare that the research was conducted in the absence of any commercial or financial relationships that could be construed as a potential conflict of interest.

## References

[B1] Aasa U. Svartholm I. Andersson F. Berglund L. (2017). Injuries among weightlifters and powerlifters: a systematic review. *Br. J. Sports Med.* 51 211–219. 10.1136/bjsports-2016-096037 27707741

[B2] Aasheim C. Stavenes H. Andersson S. H. Engbretsen L. Clarsen B. (2018). Prevalence and burden of overuse injuries in elite junior handball. *BMJ Open Sport Exerc. Med.* 4:e000391. 10.1136/bmjsem-2018-000391 30018791 PMC6045727

[B3] Adiele D. Morgan G. P. (2018). Prevalence of musculoskeletal injuries in males and females practicing swimming from higher school of Zimbabwe. *Am. J. Sports Sci.* 6 8–11. 10.11648/j.ajss.20180601.12

[B4] Airaksinen O. Brox J. I. Cedraschi C. Hildebrandt J. Klaber-Moffett J. Kovacs F. (2006). Chapter 4. European guidelines for the management of chronic nonspecific low back pain. *Eur. Spine J.* 15 S192–S300. 10.1007/s00586-006-1072-1 16550448 PMC3454542

[B5] Almeida G. P. L. de Souza V. L. Sano S. S. Saccol M. F. Cohen M. (2012). Comparison of hip rotation range of motion in judo athletes with and without history of low back pain. *Man. Ther.* 17 231–235. 10.1016/j.math.2012.01.004 22281524

[B6] Alricsson M. Björklund G. Cronholm M. Olsson O. Viklund P. Svantesson U. (2016). Spinal alignment, mobility of the hip and thoracic spine and prevalence of low back pain in young elite cross-country skiers. *J. Exerc. Rehab.* 12 21–28. 10.12965/jer.150255 26933656 PMC4771149

[B7] Alzahrani H. Shirley D. Cheng S. W. M. Mackey M. Stamatakis E. (2019). Physical activity and chronic back conditions: a population-based pooled study of 60,134 adults. *J. Sport Health Sci.* 8 386–393. 10.1016/j.jshs.2019.01.003 31333893 PMC6620421

[B8] Angoules A. G. Dionyssiotis Y. Angoules G. A. Balakatounis K. C. Panou A. Papathanasiou J. (2018). An epidemiological study of non-specific low back pain in non-professional female greek classic ballet dancers. *Folia Med.* 60 248–253. 10.1515/folmed-2017-0087 30355814

[B9] Anza R. Denis M. Silva M. F. (2013). Analysis of physical fitness, anthropometry and prevalence of musculoskeletal symptoms in the youth volleyball category. *Rev. Bras. Med. Esporte* 19 62–65.

[B10] Armstrong R. Hall B. J. Doyle J. Waters E. (2011). Cochrane update. ‘Scoping the scope’ of a cochrane review. *J. Public Health (Oxf)* 33 147–150. 10.1093/pubmed/fdr015 21345890

[B11] Augustsson S. R. Augustsson J. Thomee R. Svantesson U. (2006). Injuries and preventive actions in elite Swedish volleyball. *Scand. J. Med. Sci. Sports* 16 433–440. 10.1111/j.1600-0838.2005.00517.x 17121646

[B12] Bacon C. S. Mauger A. R. (2017). Prediction of overuse injuries in professional u18-u21 footballers using metrics of training distance and intensity. *J. Strength Cond. Res.* 31 3067–3076. 10.1519/JSC.0000000000001744 27930446

[B13] Bahr R. Reeser J. C. (2003). Injuries among world-class professional beach volleyball players: the federation internationale de volleyball beach volleyball injury study. *Am. J. Sports Med.* 31 119–125. 10.1177/03635465030310010401 12531768

[B14] Ball J. R. Harris C. B. Lee J. Vives M. J. (2019). Lumbar spine injuries in sports: review of the literature and current treatment recommendations. *Sports Med. Open* 5:26. 10.1186/s40798-019-0199-7 31236714 PMC6591346

[B15] Banister E. W. Calvert T. W. Savage M. V. Bach T. (1975). A systems model of training for athletic performance. *Austr. J. Sports Med. Exerc. Sci*. 7 57–61.

[B16] Baranto A. Hellström M. Nyman R. Lundin O. Swärd L. (2006). Back pain and degenerative abnormalities in the spine of young elite divers. *Knee Surg. Sport Tr. A* 14 907–914. 10.1007/s00167-005-0032-3 16416326

[B17] Belavý D. L. Quittner M. J. Ridgers N. Ling Y. Connell D. Rantalainen T. (2017). Running exercise strengthens the intervertebral disc. *Sci. Rep.* 7:45975. 10.1038/srep45975 28422125 PMC5396190

[B18] Bere T. Kruczynski J. Veintimilla N. Hamu Y. Bahr R. (2015). Injury risk is low among world-class volleyball players: 4-year data from the FIVB injury surveillance system. *Br. J. Sports Med.* 49 1132–1137. 10.1136/bjsports-2015-094959 26194501 PMC4552924

[B19] Bovens A. M. P. Janssen G. M. E. Vermeer H. G. W. Hoeberigs J. H. Janssen M. P. E. Verstappen F. T. J. (1989). Occurrence of running injuries in adults following a supervised training program. *Int. J. Sport Med.* 10 186–190. 10.1055/s-2007-1024970 2599739

[B20] Bowen L. Gross A. S. Gimpel M. Li F. X. (2017). Accumulated workloads and the acute:chronic workload ratio relate to injury risk in elite youth football players. *Br. J. Sports Med*. 51 452–459. 10.1136/bjsports-2015-095820 27450360 PMC5460663

[B21] Bromley S. J. Drew M. K. Talpey S. McIntosh A. S. Finch C. F. (2018). A systematic review of prospective epidemiological research into injury and illness in Olympic combat sport. *Br. J. Sports Med.* 52 8–16. 10.1136/bjsports-2016-097313 28954799

[B22] Caine D. J. Nassar L. (2005). Gymnastics injuries. *Med. Sport Sci.* 48 18–58. 10.1159/000084282 16247252

[B23] Catalá M. M. Schroll A. Laube G. Arampatzis A. (2018). Muscle strength and neuromuscular control in low-back pain: elite athletes versus general population. *Front. Neurosci.* 12:436. 10.3389/fnins.2018.00436 30018531 PMC6037821

[B24] Coenen P. Smith A. Paananen M. O’Sullivan P. Beales D. Straker L. (2017). Trajectories of low back pain from adolescence to young adulthood. *Arthritis Care Res.* 69 403–412. 10.1002/acr.22949 27273901

[B25] Cole M. H. Grimshaw P. N. (2016). The biomechanics of the modern golf swing: implications for lower back injuries. *Sports Med.* 46 339–351. 10.1007/s40279-015-0429-1 26604102

[B26] Cross M. J. Williams S. Trewartha G. Kemp S. P. Stokes K. A. (2016). The influence of in-season training loads on injury risk in professional rugby union. *Int. J. Sports Physiol. Perform.* 11 350–355. 10.1123/ijspp.2015-0187 26309331

[B27] Daniels J. M. Arguelles C. Gleason C. Dixon W. H. (2020). Back injuries. *Prim. Care* 47 147–164. 10.1016/j.pop.2019.10.008 32014131

[B28] Dennis R. J. Finch C. F. Farhart P. J. (2005). Is bowling workload a risk factor for injury to Australian junior cricket fast bowlers? *Br. J. Sports Med*. 39 843–846. 10.1136/bjsm.2005.018515 16244195 PMC1725068

[B29] Ekstrand J. (1982). *Football Injuries and Their Prevention.* Medical Dissertation No 130. Linköping: Linköping University.

[B30] Fanchini M. Rampinini E. Riggio M. Coutts A. Pecci C. McCall A. (2018). Despite association, the acute:chronic work load ratio does not predict non-contact injury in elite footballers. *Sci. Med. Football* 2 108–114. 10.1080/24733938.2018.1429014

[B31] Farahbakhsh F. Akbari-Fakhrabadi M. Shariat A. Cleland J. A. Farahbakhsh F. Seif-Bargh T. (2018). Neck pain and low back pain in relation to functional disability in different sport activities. *J. Exerc. Rehabil.* 14 509–515. 10.12965/jer.1836220.110 30018941 PMC6028206

[B32] Fett D. Trompeter K. Platen P. (2017). Back pain in elite sports: a cross-sectional study on 1114 athletes. *PLoS One* 12:e0180130. 10.1371/journal.pone.0180130 28662110 PMC5491135

[B33] Fett D. Trompeter K. Platen P. (2019). Prevalence of back pain in a group of elite athletes exposed to repetitive overhead activity. *PLoS One* 14:e0210429. 10.1371/journal.pone.0210429 30677044 PMC6345455

[B34] Foster C. Snyder A. Welsh R. (1999). “Monitoring of training, warm up, and performance in athletes,” in *Overload, Performance Incompetence and Regeneration in Sport*, eds Lehmann M. Foster C. Gastmann U. Keizer H. Steinacker J. M. (New York, NY: Kluwer Academic Plenum), 43–51. 10.1007/978-0-585-34048-7_4

[B35] Gabbett T. J. (2004a). Influence of training and match intensity on injuries in rugby league. *J. Sports Sci.* 22 409–417. 10.1080/02640410310001641638 15160594

[B36] Gabbett T. J. (2004b). Reductions in pre-season training loads reduce training injury rates in rugby league players. *Br. J. Sports Med.* 38 743–749. 10.1136/bjsm.2003.008391 15562171 PMC1725000

[B37] Gabbett T. J. (2016). The training injury prevention paradox: should athletes be training smarter and harder? *Br. J. Sports Med.* 50 273–280. 10.1136/bjsports-2015-095788 26758673 PMC4789704

[B38] Gabbett T. J. Domrow N. (2005). Risk factors for injury in subelite rugby league players. *Am. J. Sports Med.* 33 428–434. 10.1177/0363546504268407 15716260

[B39] Gabbett T. J. Domrow N. (2007). Relationships between training load, injury, and fitness in sub-elite collision sport athletes. *J. Sports Sci.* 25 1507–1519. 10.1080/02640410701215066 17852696

[B40] Gabbett T. J. Ullah S. (2012). Relationship between running loads and soft-tissue injury in elite team sport athletes. *J. Strength Cond. Res.* 26 953–960. 10.1519/JSC.0b013e3182302023 22323001

[B41] Gabbett T. J. Ullah S. Finch C. F. (2012). Identifying risk factors for contact injury in professional rugby league players—application of a frailty model for recurrent injury. *J. Sci. Med. Sport* 15 496–504. 10.1016/j.jsams.2012.03.017 22748762

[B42] Gastin P. B. Meyer D. Huntsman E. Cook J. (2015). Increase in injury risk with low body mass and aerobic-running fitness in elite Australian football. *Int. J. Sports Physiol. Perform.* 10 458–463. 10.1123/ijspp.2014-0257 25365588

[B43] Goldstein J. D. Berger P. E. Windler G. E. Jackson D. W. (1991). Spine injuries in gymnasts and swimmers: an epidemiologic investigation. *Am. J. Sports Med.* 19 463–468. 10.1177/036354659101900507 1962710

[B44] Goosey-Tolfrey V. (2010). Supporting the paralympic athlete: focus on wheeled sports. *Disabil. Rehabil.* 32 2237–2243. 10.3109/09638288.2010.491577 20528446

[B45] Graw B. P. Wiesel S. W. (2008). Low back pain in the aging athlete. *Sports Med. Arthrosc. Rev.* 16 39–46. 10.1097/JSA.0b013e318163be67 18277261

[B46] Griffin A. Kenny I. C. Comyns T. M. Lyons M. (2020). The association between the acute:chronic workload ratio and injury and its application in team sports: a systematic review. *Sports Med.* 50 561–580. 10.1007/s40279-019-01218-2 31691167

[B47] Haag T. B. Mayer H. M. Schneider A. S. Rumpf M. C. Handel M. Schneider C. (2016). Risk assessment of back pain in youth soccer players. *Res. Sports Med.* 24 395–406. 10.1080/15438627.2016.1222275 27537067 PMC5152550

[B48] Haydt R. Pheasant S. Lawrence K. (2012). The incidence of low back pain in NCAA Division III female field hockey players. *Int. J. Sports Phys. Ther.* 7 296–305. 22666644 PMC3362982

[B49] Heidari J. Hasenbring M. Kleinert J. Kellmann M. (2017). Stress-related psychological factors for back pain among athletes: important topic with scarce evidence. *Eur. J. Sport Sci.* 17 351–359. 10.1080/17461391.2016.1252429 27838957

[B50] Hoskins W. Pollard H. Daff C. Odell A. Garbutt P. Mchardy A. (2009). Low back pain status in elite and semi-elite Australian football codes: a cross-sectional survey of football (soccer), Australian rules, rugby league, rugby union and non-athletic controls. *BMC Musculoskelet. Disord.* 10:38. 10.1186/1471-2474-12-158 19371446 PMC2674424

[B51] Hulin B. T. Gabbett T. J. (2019). Indeed association does not equal prediction: the never-ending search for the perfect acute:chronic workload ratio. *Br. J. Sports Med.* 53 144–145. 10.1136/bjsports-2018-099448 29886435

[B52] Hulin B. T. Gabbett T. J. Blanch P. Chapman P. Bailey D. Orchard J. W. (2014). Spikes in acute workload are associated with increased injury risk in elite cricket fast bowlers. *Br. J. Sports Med.* 48 708–712. 10.1136/bjsports-2013-092524 23962877

[B53] Hulin B. T. Gabbett T. J. Caputi P. Lawson D. W. Sampson J. A. (2016a). Low chronic workload and the acute:chronic workload ratio are more predictive of injury than between-match recovery time: a two-season prospective cohort study in elite rugby league players. *Br. J. Sports Med.* 50 1008–1012. 10.1136/bjsports-2015-095364 26851288

[B54] Hulin B. T. Gabbett T. J. Lawson D. W. Caputi P. Sampson J. A. (2016b). The acute:chronic workload ratio predicts injury: high chronic workload may decrease injury risk in elite rugby league players. *Br. J. Sports Med*. 50 231–236. 10.1136/bjsports-2015-094817 26511006

[B55] Hunter J. S. (1986). The exponentially weighted moving average. *J. Qual. Technol*. 18 203–210.

[B56] Jacobson I. Tegner Y. (2007). Injuries among Swedish female elite football players: a prospective population study. *Scand. J. Med. Sci. Sports* 17 84–91. 10.1111/j.1600-0838.2006.00524.x 17305943

[B57] Jobson S. A. Passfield L. Atkinson G. Barton G. Scarf P. (2009). The analysis and utilization of cycling training data. *Sports Med.* 39 833–844. 10.2165/11317840-000000000-00000 19757861

[B58] Joeng H. S. Na Y. M. Lee S. Y. Cho Y. J. (2018). Injuries among Korean female professional golfers: a prospective study. *J. Sports Sci. Med.* 17 492–500. 30116123 PMC6090400

[B59] Jonasson P. Halldin K. Karlsson J. Thoreson O. Hvannberg J. Swärd L. (2011). Prevalence of joint-related pain in the extremities and spine in five groups of top athletes. *Knee Surg. Sport Tr. A.* 19 1540–1546. 10.1007/s00167-011-1539-4 21559845

[B60] Junior L. C. H. Costa L. O. P. Lopes A. D. (2013). Previous injuries and some training characteristics predict running-related injuries in recreational runners: a prospective cohort study. *J. Physiother.* 59 263–269. 10.1016/S1836-9553(13)70203-0 24287220

[B61] Kaneoka K. Shimizu K. Hangai M. Okuwaki T. Mamizuka N. Sakane M. (2007). Lumbar intervertebral disk degeneration in elite competitive swimmers: a case control study. *Am. J. Sports Med.* 8 1341–1345. 10.1177/0363546507300259 17405885

[B62] Koes B. W. van Tulder M. W. Peul W. C. (2007). Diagnosis and treatment of sciatica. *BMJ* 334 1313–1317. 10.1136/bmj.39223.428495.BE 17585160 PMC1895638

[B63] Koyama K. Nakazato K. Min S. K. Gushiken K. Hatakeda Y. Seo K. (2013). Radiological abnormalities and low back pain in gymnasts. *Int. J. Sports Med.* 34 218–222. 10.1055/s-0032-1316366 22972241

[B64] Kraft C. N. Pennekamp P. H. Becker U. Young M. Diedrich O. Lüring C. (2009). Magnetic resonance imaging findings of the lumbar spine in elite horseback riders: correlations with back pain, body mass index, trunk/leg-length coefficient, and riding discipline. *Am. J. Sports Med.* 11 2205–2213. 10.1177/0363546509336927 19574474

[B65] Külling F. A. Florianz H. Reepschläger B. Gasser J. Jost B. Lajtai G. (2014). High prevalence of disc degeneration and spondylolysis in the lumbar spine of professional beach volleyball players. *Orthop. J. Sports Med.* 2:2325967114528862. 10.1177/2325967114528862 26535316 PMC4555589

[B66] Lawrence J. P. Greene H. S. Grauer J. N. (2006). Back pain in athletes. *J. Am. Acad. Orthop.* 14 726–735. 10.5435/00124635-200612000-00004 17148620

[B67] Leppänen M. Pasanen K. Kujala U. M. Parkkari J. (2015). Overuse injuries in youth basketball and floorball. *Open Access J. Sports Med.* 6 173–179. 10.2147/OAJSM.S82305 26045679 PMC4447174

[B68] Lyman S. Fleisig G. S. Waterbor J. W. Funkhouser E. M. Pulley L. Andrews J. R. (2001). Longitudinal study of elbow and shoulder pain in youth baseball pitchers. *Med. Sci. Sports Exerc.* 33 1803–1810. 10.1097/00005768-200111000-00002 11689728

[B69] Macedo L. G. Saragiotto B. T. Yamato T. P. Costa L. O. P. Menezes Costa L. C. Ostelo R. W. J. G. (2016). Motor control exercise for acute non-specific low back pain. *Cochrane Database Syst. Rev.* 2:CD012085. 10.1002/14651858.CD012085 26863390 PMC8734597

[B70] Macera C. A. Pate R. R. Powell K. E. Jackson K. L. Kendrick J. S. Craven T. E. (1989). Predicting lower-extremity injuries among habitual runners. *Arch. Intern. Med.* 149 2565–2568. 10.1001/archinte.149.11.2565 2818115

[B71] Malone S. Roe M. Doran D. A. Gabbett T. J. Collins K. D. (2017a). Aerobic fitness and playing experience protect against spikes in workload: the role of the acute:chronic workload ratio on injury risk in elite Gaelic football. *Int. J. Sports Physiol. Perform.* 12 393–401. 10.1123/ijspp.2016-0090 27400233

[B72] Malone S. Roe M. Doran D. Gabbett T. J. Collins K. (2017b). High chronic training loads and exposure to bouts of maximal velocity running reduce injury risk in Gaelic football. *J. Sci. Med. Sport* 20 250–254. 10.1016/j.jsams.2016.08.005 27554923

[B73] Maselli F. Ciuro A. Mastrosimone R. Cannone M. Nicoli P. Signori A. (2015). Low back pain among Italian rowers: a cross-sectional survey. *J. Back Muskuloskelet.* 28 365–376. 10.3233/BMR-140529 25271199

[B74] McCall A. Dupont G. Ekstrand J. (2018). Internal workload and noncontact injury: a one-season study of five teams from the UEFA elite club injury study. *Br. J. Sports Med.* 52 1517–1522. 10.1136/bjsports-2017-098473 29626055

[B75] McHardy A. Pollard H. Luo K. (2007). One-year follow-up study on golf injuries in Australian amateur golfers. *Am. J. Sports Med.* 35 1354–1360. 10.1177/0363546507300188 17387218

[B76] Menaspa P. (2017). Are rolling averages a good way to assess training load for injury prevention? *Br. J. Sports Med*. 51 618–619. 10.1136/bjsports-2016-096131 27222309

[B77] Moradi V. Memari A. H. ShayestehFar M. Kordi R. (2015). Low back pain in athletes is associated with general and sport specific risk factors: a comprehensive review of longitudinal studies. *Rehabil. Res. Pract.* 2015:850184. 10.1155/2015/850184 26783465 PMC4691487

[B78] Murray E. Birley E. Twycross-Lewis R. Morrissey D. (2009). The relationship between hip rotation range of movement and low back pain prevalence in amateur golfers: an observational study. *Phys. Ther. Sport* 10 131–135. 10.1016/j.ptsp.2009.08.002 19897166

[B79] Murray N. B. Gabbett T. J. Townshend A. D. Hulin B. T. McLellan C. P. (2017). Individual and combined effects of acute and chronic running loads on injury risk in elite Australian footballers. *Scand. J. Med. Sci. Sports* 27 990–998. 10.1111/sms.12719 27418064

[B80] Muyor J. M. López-Miñarro P. A. Alacid F. (2011). Spinal posture of thoracic and lumbar spine and pelvic tilt in highly trained cyclists. *J. Sports Sci. Med.* 10 355–361. 24149883 PMC3761866

[B81] Muyor J. M. Zemková E. Chren M. (2017). Effects of Latin style professional dance on the spinal posture and pelvic tilt. *J. Back Musculoskelet. Rehabil.* 30 791–800. 10.3233/BMR-150448 28372311

[B82] Newlands C. Reid D. Parmar P. (2015). The prevalence, incidence and severity of low back pain among international-level rowers. *Br. J. Sports Med*. 49 951–956. 10.1136/bjsports-2014-093889 25645115

[B83] Nosco D. L. Kim Carpenter D. C. Zhang J. (1999). Determination of risk factors for low back pain in female adolescent volleyball players. *Int. J. Volleyball Res.* 7 33–42.

[B84] Orchard J. W. James T. Portus M. Kountouris A. Dennis R. (2009). Fast bowlers in cricket demonstrate up to 3- to 4-week delay between high workloads and increased risk of injury. *Am. J. Sports Med.* 37 1186–1192. 10.1177/0363546509332430 19346405

[B85] Pasanen K. Parkkari J. Kannus P. Rossi L. Palvanen M. Natri A. (2008). Injury risk in female floorball: a prospective one-season follow-up. *Scand. J. Med. Sci. Sports* 18 49–54. 10.1111/j.1600-0838.2007.00640.x 17490461

[B86] Peebles R. Jonas C. E. (2017). Sacroiliac joint dysfunction in the athlete: diagnosis and management. *Curr. Sports Med. Rep.* 16 336–342. 10.1249/JSR.0000000000000410 28902756

[B87] Piazza M. Di Cagno A. Cupisti A. Panicucci E. Santoro G. (2009). Prevalence of low back pain in former rhythmic gymnasts. *J. Sports Med. Phys. Fitness* 49 297–300. 19861936

[B88] Piotrowska S. E. Majchrzycki M. Rogala P. Mazurek-Sitarz M. (2017). Lower extremity and spine pain in cyclists. *Ann. Agric. Environ. Med.* 24 654–658. 10.5604/12321966.1233552 29284243

[B89] Plais N. Salzmann S. N. Shue J. Sanchez C. D. Urraza F. J. Girardi F. P. (2019). Spine injuries in soccer. *Curr. Sports Med. Rep.* 18 367–373. 10.1249/JSR.0000000000000638 31596753

[B90] Poór O. Zemková E. (2018). The effect of training in the preparatory and competitive periods on trunk rotational power in canoeists, ice-hockey players and tennis players. *Sports (Basel)* 6 E113. 10.3390/sports6040113 30304812 PMC6316482

[B91] Quarrie K. L. Alsop J. C. Waller A. E. Bird Y. N. Marshall S. W. Chalmers D. J. (2001). The New Zealand rugby injury and performance project. VI. A prospective cohort study of risk factors for injury in rugby union football. *Br. J. Sports Med.* 35 157–166. 10.1136/bjsm.35.3.157 11375873 PMC1724329

[B92] Reis F. J. Dias M. D. Newlands F. Meziat-Filho N. Macedo A. R. (2015). Chronic low back pain and disability in Brazilian jiu-jitsu athletes. *Phys. Ther. Sport* 16 340–343. 10.1016/j.ptsp.2015.02.005 26259668

[B93] Rostami M. Ansari M. Noormohammadpour P. Mansournia M. A. Kordi R. (2015). Ultrasound assessment of trunk muscles and back flexibility, strength and endurance in off-road cyclists with and without low back pain. *J. Back Musculoskelet. Rehabil.* 28 635–644. 10.3233/BMR-140559 25391328

[B94] Roussel N. De Kooning M. Schutt A. Mottram S. Truijen S. Nijs J. (2013). Motor control and low back pain in dancers. *Int. J. Sports Med.* 34 138–143. 10.1055/s-0032-1321722 22960991

[B95] Ruckstuhl L. Clénin G. (2019). Back pain and core strength in elite cycling. *Schweiz Z. Med. Traumatol.* 67 44–48.

[B96] Samuelsson K. Larsson H. Thyberg M. Gerdle B. (2001). Wheelchair seating intervention. Results from a client-centred approach. *Disabil. Rehabil.* 23 677–682. 10.1080/09638280110049900 11720118

[B97] Saraceni N. Kemp-Smith K. O’Sullivan P. Campbell A. (2017). The relationship between lead hip rotation and low back pain in golfers – a pilot investigation. *Int. J. Golf Sci.* 6 130–141. 10.1123/ijgs.2017-0014

[B98] Saragiotto B. T. Maher C. G. Yamato T. P. Costa L. O. P. Menezes Costa L. C. Ostelo R. W. J. G. (2016). Motor control exercise for chronic non-specific low-back pain. *Cochrane Database Syst. Rev.* 1 CD012004. 10.1002/14651858.CD012004 26742533 PMC8761501

[B99] Schultz S. J. Gordon S. J. (2010). Recreational cyclists: the relationship between low back pain and training characteristics. *Int. J. Exerc. Sci.* 3 79–85. 27182332 10.70252/HCFG8805 PMC4738893

[B100] Sekine C. Hirayama K. Yanagisawa O. Okubo Y. Hangai M. Imai A. (2014). Lumbar intervertebral disc degeneration in collegiate rowers. *J. Phys. Fitness Sports Med.* 3 525–530. 10.7600/jpfsm.3.525

[B101] Siewe J. Rudat J. Röllinghoff M. Schlegel U. J. Eysel P. Michael J. P. (2011). Injuries and overuse syndromes in powerlifting. *Int. J. Sports Med.* 32 703–711. 10.1055/s-0031-1277207 21590644

[B102] Smith B. E. Littlewood C. May S. (2014). An update of stabilisation exercises for low back pain: a systematic review with meta-analysis. *BMC Musculoskelet. Disord.* 15:416. 10.1186/1471-2474-15-416 25488399 PMC4295260

[B103] Snellman K. Parkkari J. Kannus P. Leppälä J. Vuori I. Järvinen M. (2001). Sports injuries in floorball: a prospective one-year follow-up study. *Int. J. Sports Med.* 22 531–536. 10.1055/s-2001-17609 11590481

[B104] Soligard T. Schwellnus M. Alonso J. M. Bahr R. Clarsen B. Dijkstra H. P. (2016). How much is too much? (Part 1) international olympic committee consensus statement on load in sport and risk of injury. *Br. J. Sports Med.* 50 1030–1041. 10.1136/bjsports-2016-096581 27535989

[B105] Taha T. Thomas S. G. (2003). Systems modelling of the relationship between training and performance. *Sports Med.* 33 1061–1073. 10.2165/00007256-200333140-00003 14599233

[B106] Tertti M. Paajanen H. Kujala U. M. Alanen A. Salmi T. T. Kormano M. (1990). Disc degeneration in young gymnasts: a magnetic resonance imaging study. *Am. J. Sports Med.* 18 206–208. 10.1177/036354659001800216 2140492

[B107] Tesarz J. Gerhardt A. Schommer K. Treede R. D. Eich W. (2013). Alterations in endogenous pain modulation in endurance athletes: an experimental study using quantitative sensory testing and the cold-pressor task. *Pain* 154 1022–1029. 10.1016/j.pain.2013.03.014 23657118

[B108] Tesarz J. Schuster A. K. Hartmann M. Gerhardt A. Eich W. (2012). Pain perception in athletes compared to normally active controls: a systematic review with meta-analysis. *Pain* 153 1253–1262. 10.1016/j.pain.2012.03.005 22607985

[B109] Thoreson O. Ekström L. Hansson H. A. Todd C. Witwit W. Aminoff A. S. (2017a). The effect of repetitive flexion and extension fatigue loading on the young porcine lumbar spine, a feasibility study of MRI and histological analyses. *J. Exp. Orthop.* 4:16. 10.1186/s40634-017-0091-7 28500483 PMC5429315

[B110] Thoreson O. Kovac P. Swärd A. Agnvall C. Todd C. Baranto A. (2017b). Back pain and MRI changes in the thoraco-lumbar spine of young elite Mogul skiers. *Scand. J. Med. Sci. Sports* 27 983–989. 10.1111/sms.12710 27367529

[B111] Trainor T. J. Wiesel S. W. (2002). Epidemiology of back pain in the athlete. *Clin. Sports Med.* 21 93–103. 10.1016/s0278-5919(03)00059-0 11877875

[B112] Trompeter K. Fett D. Platen P. (2017). Prevalence of back pain in sports: a systematic review of the literature. *Sports Med.* 47 1183–1207. 10.1007/s40279-016-0645-3 28035587 PMC5432558

[B113] Trompeter K. Fett D. Platen P. (2019). Back pain in rowers: a cross-sectional study on prevalence, pain characteristics and risk factors. *Sportverletz Sportschaden.* 33 51–59. 10.1055/a-0648-8387 30419587

[B114] Tunås P. Nilstad A. Myklebust G. (2015). Low back pain in female elite football and handball players compared with an active control group. *Knee Surg. Sports Traumatol. Arthrosc.* 23 2540–2547. 10.1007/s00167-014-3069-3 24839041

[B115] van Hilst J. Hilgersom N. F. Kuilman M. C. Kuijer P. P. F. M. Frings-Dresen M. H. W. (2015). Low back pain in young elite field hockey players, football players and speed skaters: prevalence and risk factors. *J. Back Musculoskelet.* 28 67–73. 10.3233/BMR-140491 24968798

[B116] van Middelkoop M. Kolkman J. van Ochten J. Bierma-Zeinstra S. M. A. Koes B. W. (2007). Course and predicting factors of lower-extremity injuries after running a marathon. *Clin. J. Sport Med.* 17 25–30. 10.1097/JSM.0b013e3180305e4d 17304002

[B117] van Middelkoop M. Kolkman J. van Ochten J. Bierma−Zeinstra S. M. A. Koes B. W. (2008). Risk factors for lower extremity injuries among male marathon runners. *Scand. J. Med. Sci. Sports* 18 691–697. 10.1111/j.1600-0838.2007.00768.x 18266787

[B118] Villavicencio A. T. Burneikienë S. Hernández T. D. Thramann J. (2006). Back and neck pain in triathletes. *Neurosurg. Focus* 21 1–7. 10.3171/foc.2006.21.4.8 17112197

[B119] Wilber C. A. Holland G. J. Madison R. E. Loy S. F. (1995). An epidemiological analysis of overuse injuries among recreational cyclists. *Int. J. Sport Med.* 16 201–206. 10.1055/s-2007-972992 7649713

[B120] Williams S. West S. Cross M. J. Stokes K. A. (2017). Better way to determine the acute:chronic workload ratio? *Br. J. Sports Med.* 51 209–210. 10.1136/bjsports-2016-096589 27650255

[B121] Windt J. Gabbett T. J. Ferris D. Khan K. M. (2017). Training load–injury paradox: is greater preseason participation associated with lower in-season injury risk in elite rugby league players? *Br. J. Sports Med.* 51 645–650. 10.1136/bjsports-2016-095973 27075963

[B122] Wirth K. Hartmann H. Mickel C. Szilvas E. Keiner M. Sander A. (2017). Core stability in athletes: a critical analysis of current guidelines. *Sports Med.* 47 401–414. 10.1007/s40279-016-0597-7 27475953

[B123] Witwit W. A. Kovac P. Sward A. Agnvall C. Todd C. Thoreson O. (2018). Disc degeneration on MRI is more prevalent in young elite skiers compared to controls. *Knee Surg. Sport Tr. A.* 26 325–332. 10.1007/s00167-017-4545-3 28409199 PMC5754419

[B124] Zemková E. Cepková A. Uvaček M. Hamar D. (2016a). A new method to assess the power performance during a lifting task in young adults. *Measurement* 91 460–467. 10.1016/j.measurement.2016.05.077

[B125] Zemková E. Cepková A. Uvaček M. Šooš L’ (2017). A novel method for assessing muscle power during the standing cable wood chop exercise. *J. Strength Cond. Res.* 31 2246–2254. 10.1519/JSC.0000000000001692 27806016

[B126] Zemková E. Hamar D. (2018). Sport-specific assessment of the effectiveness of neuromuscular training in young athletes. *Front. Physiol.* 9:264. 10.3389/fphys.2018.00264 29695970 PMC5904431

[B127] Zemková E. Jeleň M. (2019). Differentiation of the strength of back muscle contraction under fatigue: Does force feedback play a role? *J. Sport Rehabil.* 10.1123/jsr.2018-0496 [Epub ahead of print]. 31593928

[B128] Zemková E. Jeleň M. Zapletalová L. (2018a). Trunk rotational velocity in young and older adults: a role of trunk angular displacement. *Res. Phys. Educ. Sport Health* 7 103–107.

[B129] Zemková E. Miklovič P. Dunajčík A. Hamar D. (2015). Power as a parameter in the functional assessment of knee flexions and knee extensions on weight stack machines. *Measurement* 61 141–149.

[B130] Zemková E. Muyor J. M. Jeleň M. (2018b). Association of trunk rotational velocity with spine mobility and curvatures in para table tennis players. *Int. J. Sports Med.* 39 1055–1062. 10.1055/a-0752-4224 30452067

[B131] Zemková E. Poór O. Jeleň M. (2019a). Between-side differences in trunk rotational power in athletes trained in asymmetric sports. *J. Back Musculoskelet. Rehabil.* 32 529–537. 10.3233/BMR-181131 30584114

[B132] Zemková E. Poór O. Pecho J. (2019b). Peak rate of force development and isometric maximum strength of back muscles are associated with power performance during load-lifting tasks. *Am. J. Men’s Health* 13 1–8. 10.1177/1557988319828622 30819070 PMC6775675

[B133] Zemková E. Štefániková G. Muyor J. M. (2016b). Load release balance test under unstable conditions effectively discriminates between physically active and sedentary young adults. *Hum. Mov. Sci.* 48 142–152. 10.1016/j.humov.2016.05.002 27203382

